# Emerging roles of ARHGAP33 in intracellular trafficking of TrkB and pathophysiology of neuropsychiatric disorders

**DOI:** 10.1038/ncomms10594

**Published:** 2016-02-03

**Authors:** Takanobu Nakazawa, Ryota Hashimoto, Kazuto Sakoori, Yuki Sugaya, Asami Tanimura, Yuki Hashimotodani, Kazutaka Ohi, Hidenaga Yamamori, Yuka Yasuda, Satomi Umeda-Yano, Yuji Kiyama, Kohtarou Konno, Takeshi Inoue, Kazumasa Yokoyama, Takafumi Inoue, Shusuke Numata, Tohru Ohnuma, Nakao Iwata, Norio Ozaki, Hitoshi Hashimoto, Masahiko Watanabe, Toshiya Manabe, Tadashi Yamamoto, Masatoshi Takeda, Masanobu Kano

**Affiliations:** 1 Department of Neurophysiology, Graduate School of Medicine, The University of Tokyo, Tokyo 113-0033, Japan; 2 Division of Oncology, Institute of Medical Science, The University of Tokyo, Tokyo 108-8639, Japan; 3 iPS Cell-based Research Project on Brain Neuropharmacology and Toxicology, Graduate School of Pharmaceutical Sciences, Osaka University, Suita 565-0871, Japan; 4 Department of Psychiatry, Osaka University Graduate School of Medicine, Suita 565-0871, Japan; 5 Molecular Research Center for Children's Mental Development, United Graduate School of Child Development, Osaka University, Suita 565-0871, Japan; 6 Department of Molecular Neuropsychiatry, Osaka University Graduate School of Medicine, Suita 565-0871, Japan; 7 Division of Neuronal Network, Institute of Medical Science, The University of Tokyo, Tokyo 108-8639, Japan; 8 Department of Anatomy, Hokkaido University Graduate School of Medicine, Sapporo 060-8638, Japan; 9 Department of Life Science and Medical Bioscience, School of Advanced Science and Engineering, Waseda University, Tokyo 162-8480, Japan; 10 Department of Psychiatry, Course of Integrated Brain Sciences, School of Medicine, University of Tokushima, Tokushima 770-8503, Japan; 11 Department of Psychiatry, Juntendo University School of Medicine, Tokyo 113-0033, Japan; 12 Department of Psychiatry, Fujita Health University School of Medicine, Toyoake 470-1192, Japan; 13 Department of Psychiatry, Nagoya University Graduate School of Medicine, Nagoya 461-8673, Japan; 14 Laboratory of Molecular Neuropharmacology, Graduate School of Pharmaceutical Sciences, Osaka University, Suita 565-0871, Japan; 15 Cell Signal Unit, Okinawa Institute of Science and Technology Graduate University, Onna-son 904-0495, Japan

## Abstract

Intracellular trafficking of receptor proteins is essential for neurons to detect various extracellular factors during the formation and refinement of neural circuits. However, the precise mechanisms underlying the trafficking of neurotrophin receptors to synapses remain elusive. Here, we demonstrate that a brain-enriched sorting nexin, ARHGAP33, is a new type of regulator for the intracellular trafficking of TrkB, a high-affinity receptor for brain-derived neurotrophic factor. *ARHGAP33* knockout (KO) mice exhibit reduced expression of synaptic TrkB, impaired spine development and neuropsychiatric disorder-related behavioural abnormalities. These deficits are rescued by specific pharmacological enhancement of TrkB signalling in *ARHGAP33* KO mice. Mechanistically, ARHGAP33 interacts with SORT1 to cooperatively regulate TrkB trafficking. Human *ARHGAP33* is associated with brain phenotypes and reduced *SORT1* expression is found in patients with schizophrenia. We propose that ARHGAP33/SORT1-mediated TrkB trafficking is essential for synapse development and that the dysfunction of this mechanism may be a new molecular pathology of neuropsychiatric disorders.

## Abstract (web summary)

The molecular mechanisms of neurotrophin receptor trafficking are only partially understood. Here the authors show that ARHGAP33 interacts with SORT1 to regulate TrkB trafficking, the dysfunction of which impairs synapse development and leads to schizophrenia-related behavioural abnormalities in mice.

## Graphical abstract

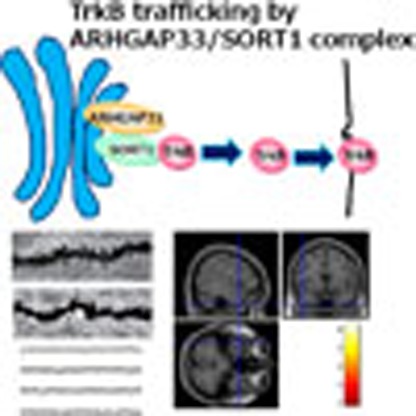

Intracellular protein trafficking is essential for cellular functions particularly in highly polarized cells such as neurons^1^. Membrane proteins are generally delivered in a polarized manner from the endoplasmic reticulum, the Golgi apparatus and the trans-Golgi network to synaptic sites^2^,^3^. Multiple classes of proteins are responsible for ensuring the specificity of sorting and trafficking^3^, including proteins of the sorting nexin (SNX) family, a large group of proteins that contain a conserved phox homology (PX) domain. Through a conserved PX domain-mediated interaction with phosphoinositides, SNX proteins are often localized to the Golgi apparatus and endosomes, where they regulate the exiting and sorting of membrane proteins^4^. ARHGAP33 (also known as SNX26, TCGAP or NOMA-GAP; hereafter ARHGAP33)^5^,^6^,^7^,^8^,^9^ and ARHGAP32 (also known as p250GAP and PX-RICS; hereafter ARHGAP32)^10^,^11^,^12^ represent a unique subfamily of SNX proteins that have a RhoGTPase-activating protein (RhoGAP) domain (for a review, see ref. 13). These SNX proteins are highly enriched in the brain, but it remains unclear whether and how they are involved in protein sorting and trafficking in neurons and contribute to higher brain functions.

TrkB is a high-affinity receptor for brain-derived neurotrophic factor (BDNF) that plays important roles in the neuronal development, establishment and maintenance of synapses, regulation of synaptic transmission and plasticity, and memory formation^14^,^15^,^16^. TrkB function is regulated by multiple steps, including transcriptional, translational and post-translational mechanisms^14^,^15^. Among them, a critical step is the proper trafficking of TrkB from the soma to the distal compartments of axons and dendrites^14^,^15^, but the mechanisms of TrkB trafficking remain unclear.

In the present study, we show that ARHGAP33 regulates the trafficking of TrkB to synaptic sites. Consistent with the role of TrkB in synapse maintenance and function^14^,^15^,^16^, *ARHGAP33* KO mice have impaired spine morphogenesis and exhibit behavioural deficits. Mechanistically, ARHGAP33 functions cooperatively with sortilin (SORT1), a modulator of intracellular protein trafficking^17^, to regulate TrkB trafficking to synapses. Interestingly, correlated decreases in *ARHGAP33* and *SORT1* expression levels are observed in the peripheral lymphocytes of schizophrenia patients. Furthermore, human *ARHGAP33* is associated with brain phenotypes of patients with schizophrenia. We argue that ARHGAP33/SORT1-mediated TrkB trafficking is crucial for synapse development and that its disruption may lead to pathogenesis of neuropsychiatric disorders.

## Results

### Decreased surface expression of TrkB in *ARHGAP33* KO mice

**Figure 1:**
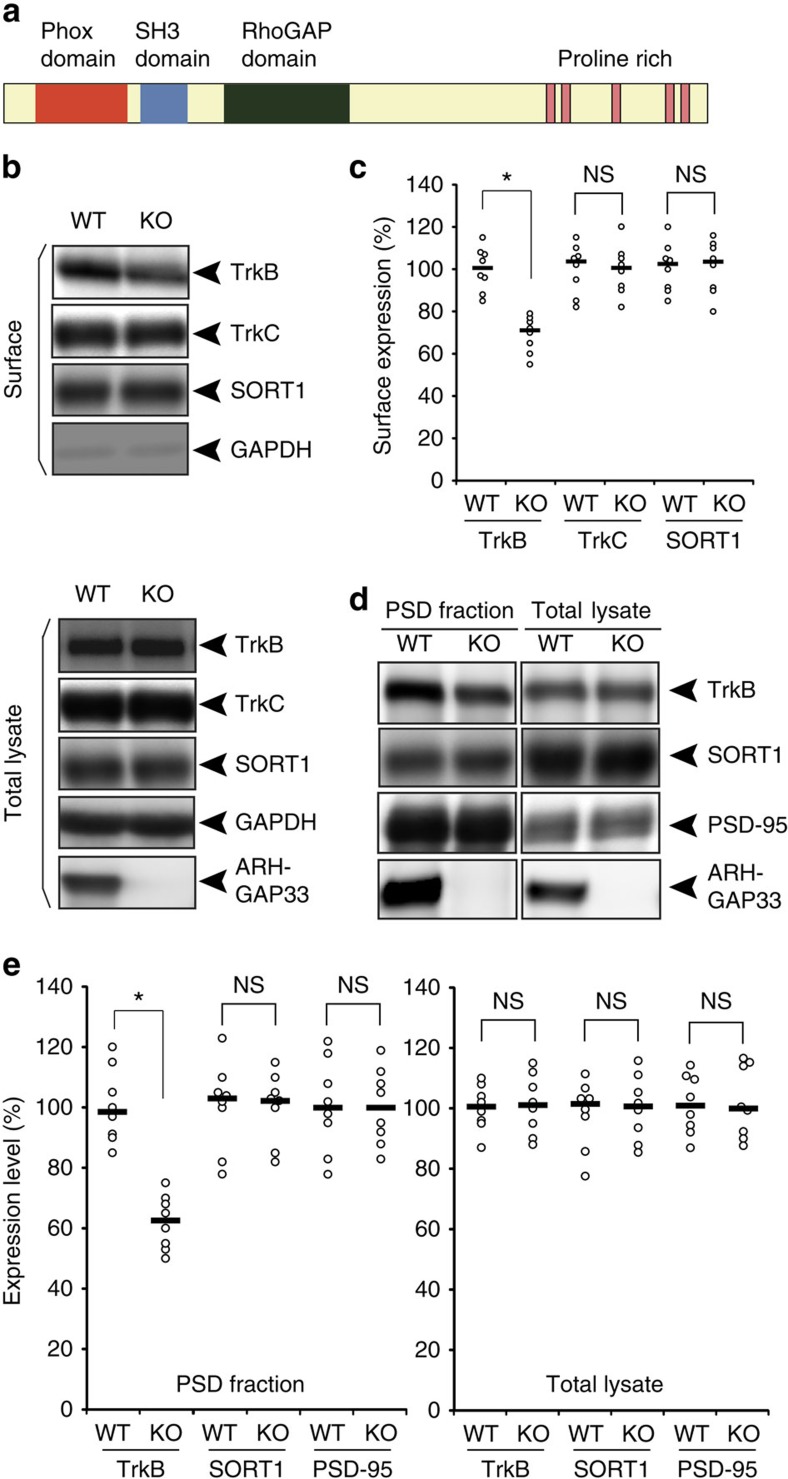
Impaired TrkB trafficking to the cell surface at synapses in *ARHGAP33* KO mice. (**a**) Protein structure of a brain-enriched SNX protein, ARHGAP33. ARHGAP33 has an N-terminal PX domain, an SH3 domain and a RhoGAP domain. (**b**,**c**) Decreased cell-surface expression of TrkB in *ARHGAP33* KO mice. Biotinylated cell-surface proteins (upper) and total lysates (lower) of WT and *ARHGAP33* KO neurons (14 DIV) were immunoblotted with anti-TrkB, anti-TrkC, anti-SORT1, anti-GAPDH and anti-ARHGAP33 antibodies. (**b**) Representative blots. (**c**) Quantification of surface expression (each, *n*=8; TrkB, *P*=7.8 × 10^−4^; TrkC and SORT1, *P*>0.05; Mann–Whitney *U*-test). The expression levels of TrkB, TrkC and SORT1 in *ARHGAP33* KO neurons were normalized to those in WT neurons (The averaged WT values were set to 100%). (**d**,**e**) Decreased TrkB in the isolated PSD fraction of *ARHGAP33* KO mice. The isolated PSD fraction and total lysates of WT and *ARHGAP33* KO mice were immunoblotted with anti-TrkB, anti-PSD-95, anti-SORT1, and anti-ARHGAP33 antibodies. Representative blots (**d**). Quantification for the isolated PSD fraction (each, *n*=8, TrkB, *P*=7.7 × 10^−4^; SORT1 and PSD-95, *P*>0.05; Mann–Whitney *U*-test) and for the total lysate (each, *n*=8, *P*>0.05; Mann–Whitney *U*-test; **e**) The expression levels of TrkB, SORT1 and PSD-95 in the PSD fraction and total lysate from *ARHGAP33* KO mice were normalized to those from WT mice (The averaged WT values were set to 100%). Note that the amounts of PSD-95 and SORT1 in the isolated PSD fraction from *ARHGAP33* KO mice were not significantly different from those in the fraction from WT mice. *P<0.05. NS, not significant. Bars show median values. All western blots show representative results from eight independent experiments performed using different mice.

ARHGAP33 is a unique, multidomain protein containing the RhoGAP, SH3 and PX domains (Fig. 1a) and is highly expressed in the brain, especially in the cortex, hippocampus, caudate-putamen and olfactory bulb (Supplementary Fig. 1)^7^. To examine ARHGAP33 functions *in vivo*, we generated *ARHGAP33* KO mice. The KO mice were born according to Mendelian genetics, exhibited normal growth and did not show severe abnormalities (Supplementary Fig. 2). The gross anatomy and cytoarchitecture of the *ARHGAP33* KO brains were apparently normal (Supplementary Fig. 2). The roles of ARHGAP33 in the adult brain have not been investigated, but given that ARHGAP33 is an SNX protein, ARHGAP33 may regulate the trafficking of surface proteins. To examine this possibility, we performed a cell-surface biotinylation assay in dissociated hippocampal neurons from *ARHGAP33* KO mice and analysed the cell-surface expression levels of various neural receptors. We found that the expression level of cell-surface-localized TrkB, but not that of total TrkB, was significantly decreased in neurons from *ARHGAP33* KO mice compared with those from wild-type (WT) mice (*P*=7.8 × 10^−4^; Fig. 1b,c). In contrast, the cell-surface expression level of another Trk family member, TrkC, was unchanged (*P*>0.05; Fig. 1b,c). As another control, we examined GAPDH expression in the cell-surface fraction. However, it was hardly detectable (Fig. 1b,c), presumably because of the dominant cytoplasmic localization of GAPDH. Since TrkB localizes to the postsynaptic density (PSD) of excitatory synapses that contains receptors and their associated signalling and scaffolding proteins^2^,^18^, we then examined TrkB expression in the PSD fraction. We found that the expression of TrkB but not PSD-95 in the PSD fraction was also decreased in *ARHGAP33* KO mice compared with WT mice (*P*=7.7 × 10^−4^; Fig. 1d,e). We performed the same analyses for SORT1, a modulator of intracellular protein trafficking^17^ that regulates the sorting of TrkB at the Golgi apparatus^19^,^20^. Although SORT1 was reported to be enriched in the Golgi apparatus^17^, we detected SORT1 in the cell-surface fraction (Fig. 1b,c) and the PSD fraction (Fig. 1d,e). We found no significant differences in SORT1 expression in these cell fractions as well as in the total lysate between WT and *ARHGAP33* KO mice.

### Impaired spine morphology in *ARHGAP33* KO hippocampus

**Figure 2:**
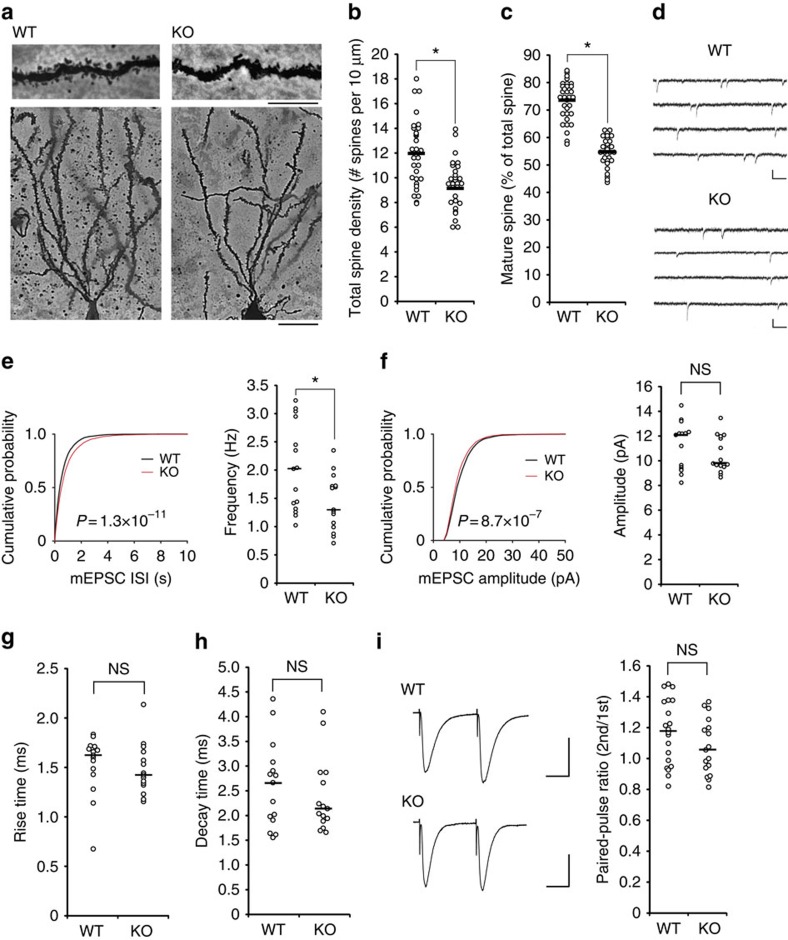
Impaired spine development in *ARHGAP33* KO mice. (**a**–**c**) Decreased total and mature spine densities in *ARHGAP33* KO mice. Examples of Golgi staining of granule cells in the hippocampal dentate gyri from 12-week-old *ARHGAP33* KO and WT mice (**a**). Scale bars, 10 μm. Z-stacks were imaged, and individual spines were measured (WT, *n*=33 cells, KO, *n*=29 cells; each *n*=4 mice; total spine density, *P*=4.4 × 10^−5^; percentage of mature spines, *P*=7.7 × 10^−17^, one-way ANOVA; **b**,**c**). *P<0.05. Bars show mean values. Note that dendritic protrusions with widths larger than half their length were classified as mature spines^49^. (**d**–**h**) Decreased mEPSC frequency and amplitude in *ARHGAP33* KO dentate gyrus granule cells. Representative traces of mEPSCs obtained from hippocampal slices of 12-week-old WT and *ARHGAP33* KO mice (**d**). Scale bar, 10 pA, 100 ms. Cumulative probability plot and summary of average mEPSC frequency and amplitude of the same neurons (**e**–**h**). mEPSC frequency (cumulative probability plot, WT, *n*=2,937 events, KO, *n*=2,139 events, *P*=1.3 × 10^−11^, Kolmogorov–Smirnov test; average (scatterplot), WT, *n*=15 cells, KO, *n*=15 cells, *P*=0.017, Mann–Whitney *U*-test; **e**); mEPSC amplitude (cumulative probability plot, WT, *n*=2,937 events, KO, *n*=2,139 events, *P*=8.7 × 10^−7^, Kolmogorov–Smirnov test; average (scatterplot), WT, *n*=15 cells, KO, *n*=15 cells, *P*>0.05, Mann–Whitney *U*-test; **f**); rise time (**g**) and decay time (**h**; WT, *n*=15 cells, KO, *n*=15 cells, *P*>0.05, Mann–Whitney *U*-test). *P<0.05. Bars in the summary plots show median values. (**i**) No change in the paired-pulse ratio of evoked EPSCs from *ARHGAP33* KO dentate gyrus granule cells. Representative traces of EPSCs (left). Scale bars, 100 pA, 20 ms. Summary graph showing the ratio of the second to the first EPSC amplitude (WT, *n*=20 cells; KO, *n*=17 cells, *P*>0.05, Mann–Whitney *U*-test; right). NS, not significant.

Given that synaptic TrkB signalling modulates the density and morphology of dendritic spines, which underlies higher brain functions such as learning and memory^14^,^15^,^21^,^22^, decreased TrkB expression at synapses may affect spine morphogenesis in *ARHGAP33* KO mice. To examine this possibility, we performed a Golgi impregnation analysis of adult brains from *ARHGAP33* KO and WT mice (Fig. 2a). We observed a decreased total spine density and a reduced proportion of mature spines in the primary dendrites of hippocampal dentate gyrus granule cells in *ARHGAP33* KO mice (total spine density, *P*=4.4 × 10^−5^; percentage of mature spines, *P*=7.7 × 10^−17^; Fig. 2b,c). We next evaluated whether the reduction in spine density in *ARHGAP33* KO mice was accompanied by a functional reduction in excitatory synaptic transmission. We performed whole-cell voltage clamp recordings to assess miniature excitatory postsynaptic currents (mEPSCs) in dentate gyrus granule cells in acute brain slices. We observed a significant decrease in the mEPSC frequency in *ARHGAP33* KO mice compared with WT mice (cumulative, *P*=1.3 × 10^−11^; average, *P*=0.017; Fig. 2d,e). We also found that the mEPSC amplitude was slightly decreased in *ARHGAP33* KO mice (cumulative, *P*=8.7 × 10^−7^; average, *P*>0.05; Fig. 2d,f). No significant changes in the rise time or decay time constant were observed (*P*>0.05; Fig. 2g,h). Furthermore, no significant differences in the paired-pulse amplitude ratio of evoked AMPA receptor-mediated EPSCs were observed between *ARHGAP33* KO and WT mice (*P*>0.05; Fig. 2i), indicating that the observed decrease in the mEPSC frequency in *ARHGAP33* KO mice results from a decrease in the number of functional synapses rather than a decrease in the probability of presynaptic release. Although the recordings may miss significant amounts of smaller synaptic events, particularly in *ARHGAP33* KO neurons, these results demonstrate that ARHGAP33 regulates the number of functional excitatory synapses.

### Behavioural deficits in *ARHGAP33* KO mice

**Figure 3:**
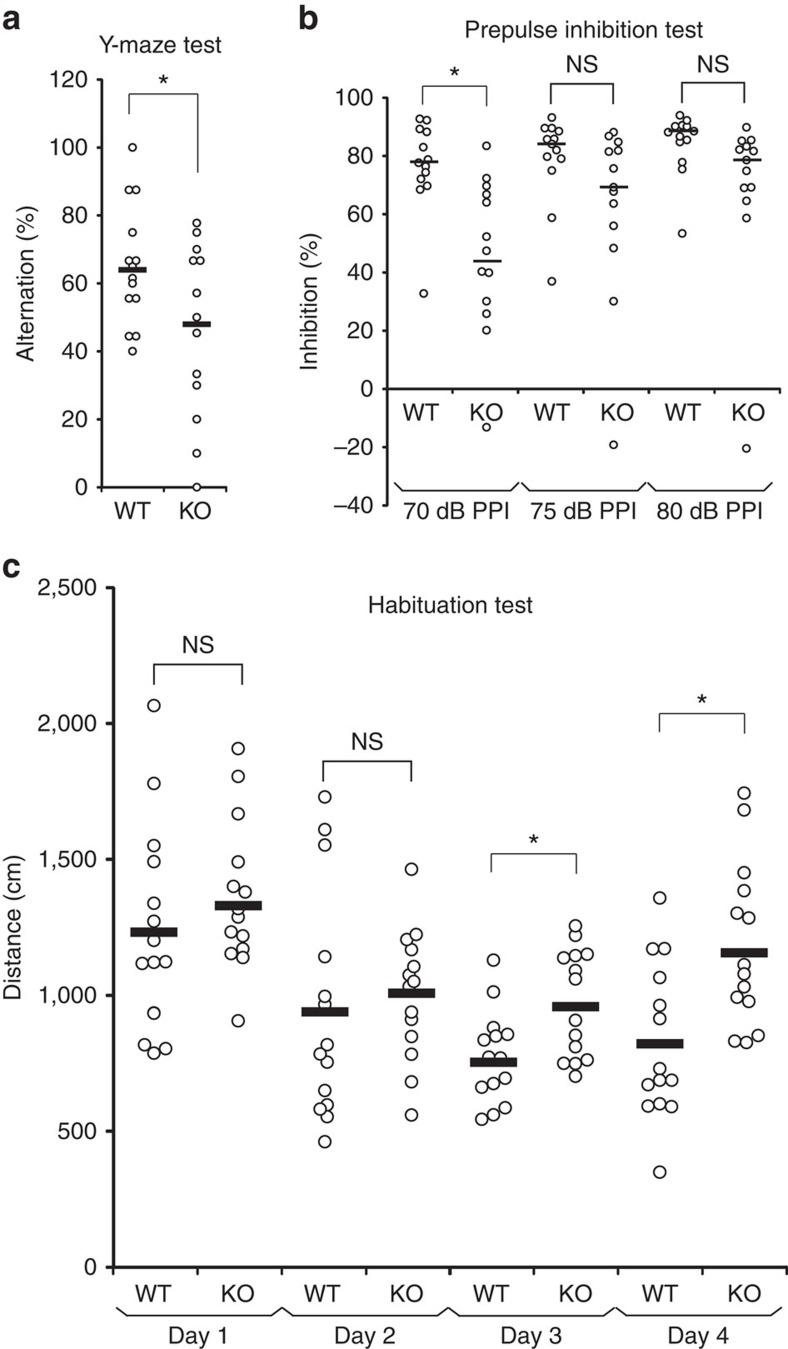
Behavioural abnormalities in *ARHGAP33* KO mice. (**a**) Impaired spontaneous alternations of *ARHGAP33* KO mice during the Y-maze test (WT, *n*=14, KO, *n*=13, F_1,25_=5.18, *P*=0.031, one-way ANOVA). *P<0.05. Bars show mean values. (**b**) Impaired PPI of *ARHGAP33* KO mice (each *n*=13, *P*=0.046, Friedman test followed by Scheffe tests). *P<0.05. NS, not significant. Bars show median values. (**c**) Impaired open field habituation of *ARHGAP33* KO mice during the openfield habituation test (each *n*=14, genotype effect, F_1,26_=5.08, *P*=0.033, two-way ANOVA with repeated measures; day 3, *P*=1.3 × 10^−4^, day 4, *P*=7.0 × 10^−4^, Tukey–Kramer *post hoc* tests). *P<0.05. NS, not significant. Bars show mean values.

*ARHGAP33* KO mice exhibited normal basic neural functions, including motor coordination and spontaneous locomotor activity (Supplementary Fig. 3a,b). We then performed a battery of behavioural tests to evaluate sensory function, cognition, depression and anxiety. The performances of *ARHGAP33* KO mice in the contextual fear conditioning, auditory fear conditioning, water maze, elevated plus-maze and startle amplitude tests were normal (Supplementary Fig. 3c–g). In contrast, we found that *ARHGAP33* KO mice exhibited abnormal behaviour in the Y-maze, prepulse inhibition (PPI) and open-field habituation tests. The Y-maze test is a spatial working memory task based on the natural tendency of mice to alternate the choice of maze arms. *ARHGAP33* KO mice exhibited reduced alternation (*P*=0.031; Fig. 3a), suggesting a spatial working memory deficit. The PPI test provides an operational measure of sensorimotor gating, in which a weaker prepulse inhibits the reaction to a subsequent strong startling pulse. Before the test, we measured the startle amplitude of control and *ARHGAP33* KO mice and determined that the startle responses of the two groups of mice were not significantly different (*P*>0.05; Supplementary Fig. 3h). In *ARHGAP33* KO mice, the percent of the PPI of the startle reflex was significantly lower than that of WT mice, indicating impaired PPI of the startle reflex (*P*=0.046; Fig. 3b). The open-field habituation test assesses the ability of mice to learn and remember an open-field chamber^23^. Although WT mice habituated to the open field (that is, decreased exploratory activity with increased exposure to the chamber over multiple trials), *ARHGAP33* KO mice did not exhibit clear habituation (day 3, *P*=1.3 × 10^−4^; day 4, *P*=7.0 × 10^−4^; Fig. 3c). These behavioural abnormalities are often observed in both mouse models of and human patients with neuropsychiatric disorders^24^,^25^.

### Enhancing TrkB signalling rescues the deficits

**Figure 4:**
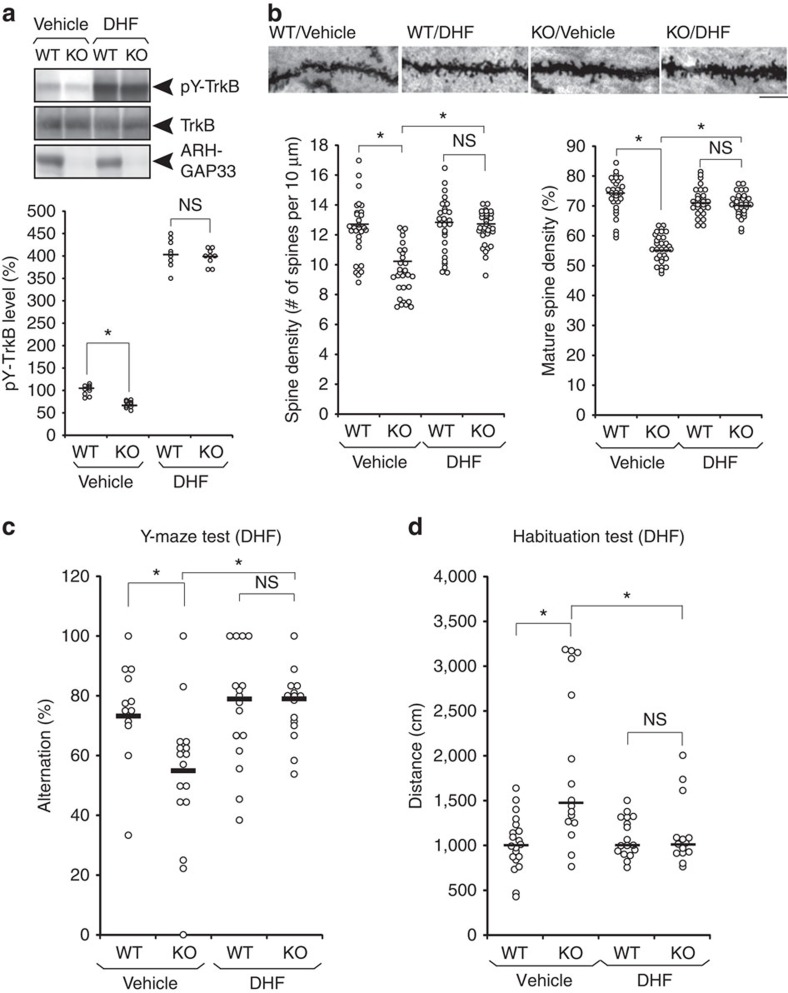
Rescue of the impaired spine development and behavioural abnormalities in *ARHGAP33* KO mice via TrkB activation in adulthood. (**a**) Activation of TrkB by 7,8-DHF injection (12.5 mg kg^−1^). TrkB-immunoprecipitates and hippocampal lysates were immunoblotted with the indicated antibodies. Representative blots (upper) and quantification of phospho-TrkB levels (lower; each *n*=9, corrected *P*=0.0014, Mann–Whitney *U*-test with the Ryan's correction). The pY-TrkB levels of vehicle-KO, DHF-WT and DHF-KO were normalized to that of vehicle-WT (The averaged vehicle-WT value was set to 100%). Bars show median values. (**b**) Rescue of the decreased number of spines in *ARHGAP33* KO neurons after 2 weeks of daily treatment with 7,8-DHF. Examples of dentate granule Golgi staining (upper). Scale bars, 10 μm. Quantification of the total spine density and the percentage of mature spines (vehicle-WT, *n*=29 cells, vehicle-KO, *n*=30, DHF-WT, *n*=28 cells, DHF-KO, *n*=31 cells, each *n*=4 mice; spine density, F_1,114_=19.7, *P*=2.1 × 10^−5^, two-way ANOVA; *P*=9.7 × 10^−10^ (vehicle-WT versus vehicle-KO), *P*=3.6 × 10^−10^ (vehicle-KO versus DHF-KO), Tukey–Kramer *post hoc* tests; mature spine density, F_1,114_=83.7, *P*=2.6 × 10^−15^, two-way ANOVA; *P*=7.3 × 10^−26^ (vehicle-WT versus vehicle-KO), *P*=4.9 × 10^−21^ (vehicle-KO versus DHF-KO), Tukey–Kramer *post hoc* tests; lower). Bars show mean values. (**c**) Rescue of the impaired working memory in *ARHGAP33* KO mice during the Y-maze test after treatment with 7,8-DHF (vehicle-WT, *n*=12, vehicle-KO, *n*=16, DHF-WT, *n*=16, DHF-KO, *n*=15, DHF treatment × genotype interaction, F_1,55_=4.60, *P*=0.036, two-way ANOVA; *P*=4.6 × 10^−3^ (vehicle-WT versus vehicle-KO), *P*=1.1 × 10^−3^ (vehicle-KO versus DHF-KO), Tukey–Kramer *post hoc* tests). Bars show mean values. (**d**) Rescue of the impaired open-field habituation in *ARHGAP33* KO mice after treatment with 7,8-DHF on the test day (day 4; vehicle-WT, *n*=20, vehicle-KO, *n*=16, DHF-WT, *n*=17, DHF-KO, *n*=14, corrected *P*=0.0052 (vehicle-WT versus vehicle-KO), corrected *P*=0.035 (vehicle-KO versus DHF-KO), Mann–Whitney *U*-test with the Ryan's correction). *P<0.05. NS, not significant. Bars show median values.

If the altered spine morphology and behavioural deficits in *ARHGAP33* KO mice are associated with the reduced surface TrkB expression (Fig. 1), activation of TrkB signalling should rescue these phenotypes. To test this possibility, we intraperitoneally injected 7,8-dihydroxyflavone (DHF) into *ARHGAP33* KO and WT mice. 7,8-DHF is a recently identified direct activator of TrkB *in vivo* that can cross the blood–brain barrier after intraperitoneal administration^26^,^27^. Recent studies have shown that the administration of 7,8-DHF has a profound effect on synaptic plasticity and behaviour in mice via TrkB activation^28^,^29^. In *ARHGAP33* KO mice, the level of phospho-TrkB was decreased compared with that in WT mice (Fig. 4a; corrected *P*=0.0014), which seems consistent with the decreased surface TrkB expression. 7,8-DHF administration (12.5 mg kg^−1^) increased the level of phospho-TrkB in *ARHGAP33* mice to the same extent as in WT mice (7,8-DHF-stimulated WT mice versus 7,8-DHF-stimulated *ARHGAP33* KO mice, *P*>0.05; Fig. 4a). This result suggests that the administration of an exogenous agonist can activate TrkB-mediated signalling sufficiently in *ARHGAP33* KO mice, although the surface TrkB expression levels were significantly decreased in *ARHGAP33* KO mice. To examine the effect of 7,8-DHF on spine morphogenesis, we made a daily intraperitoneal injection of 12.5 mg kg^−1^ of 7,8-DHF into mice for 14 days starting at 12 weeks of age. We found that this chronic 7,8-DHF administration successfully restored the decreased spine density and decreased proportion of mature spines in the dentate gyrus granule cells of *ARHGAP33* KO mice to the levels of WT mice (7,8-DHF WT mice versus 7,8-DHF *ARHGAP33* KO mice, *P*>0.05; Fig. 4b). Since the impaired spine morphogenesis in *ARHGAP33* KO mice can be rescued in adulthood at 12 weeks of age, ARHGAP33 may be important for the maintenance rather than the formation of spines. We then studied the behaviour of *ARHGAP33* KO mice after administration of 7,8-DHF. The chronic 7,8-DHF administration rescued the impaired working memory (that is, decreased spontaneous alternation in the Y-maze) of adult *ARHGAP33* KO mice, restoring it to the level observed in WT mice (vehicle-WT mice versus vehicle-*ARHGAP33* KO mice, *P*=4.6 × 10^−3^; vehicle *ARHGAP33* KO mice versus 7,8-DHF *ARHGAP33* KO mice, *P*=1.1 × 10^−3^; 7,8-DHF WT mice versus 7,8-DHF *ARHGAP33* KO mice, *P*>0.05; Fig. 4c). We next examined the effect of the chronic 7,8-DHF administration on the abnormal habituation of *ARHGAP33* KO mice. Similar to Fig. 3c, we found that vehicle-injected WT but not *ARHGAP33* KO mice exhibited clear habituation to the open field on day 4 (WT, day 1, 1,767±86 cm (median±s.e.m.), day 4, 996±69 cm, corrected *P*<0.05; KO, day 1, 1,880±168 cm, day 4, 1,467±221 cm, corrected *P*>0.05; Mann–Whitney *U*-test with the Ryan's correction). The chronic 7,8-DHF administration restored the abnormal habituation of adult *ARHGAP33* KO mice to the level observed in WT mice (vehicle-WT mice versus vehicle-*ARHGAP33* KO mice, corrected *P*=0.0052; vehicle *ARHGAP33* KO mice versus 7,8-DHF *ARHGAP33* KO mice, corrected *P*=0.035; 7,8-DHF WT mice versus 7,8-DHF *ARHGAP33* KO mice, *P*>0.05; Fig. 4d). These results suggest that ARHGAP33 positively regulates spine morphogenesis and the behavioural performances by enhancing TrkB expression at synaptic membranes and that the deficits observed in *ARHGAP33* KO mice may be reversible by pharmacological manipulation, even in adulthood. Regarding the PPI test, although we observed a marginal impairment of PPI in vehicle-injected *ARHGAP33* KO mice compared with vehicle-injected WT mice, this difference was not statistically significant (70 dB, vehicle-WT, 60.3±5.2%, vehicle-KO, 47.2±10.1%, *P*>0.05) and therefore it was impossible to perform the rescue experiments. We assume that the lack of difference might be attributable to the stress caused by daily intraperitoneal injection for 14 days.

### Increased TrkB at the Golgi apparatus in *ARHGAP33* KO mice

**Figure 5:**
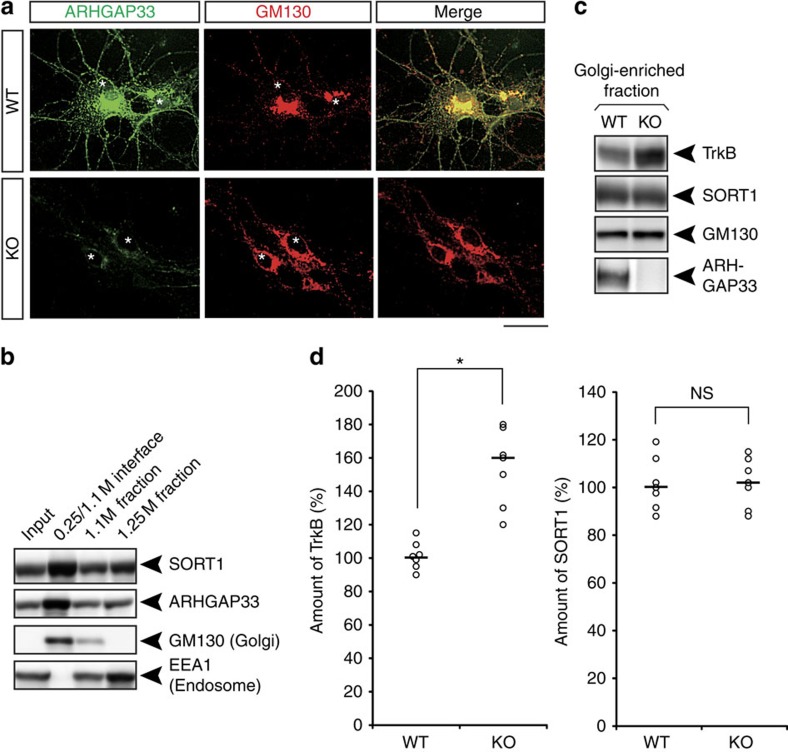
Increased TrkB at the Golgi apparatus in *ARHGAP33* KO mice. (**a**) ARHGAP33 was localized to the Golgi apparatus. Double immunostaining for ARHGAP33 and a Golgi marker, GM130, in dissociated hippocampal neurons. Scale bar, 5 μm. Asterisks indicate the nucleus of neurons. Note that ARHGAP33 immunoreactivity was not detected in the neurons from *ARHGAP33* KO mice (lower). The data are representative of three independent experiments. (**b**) ARHGAP33 and SORT1 were co-fractionated with a Golgi marker, GM130. Biochemical preparation of the Golgi membrane fraction from a mouse brain lysate with a discontinuous sucrose density gradient. Equal amounts of protein were loaded into individual lanes and probed with antibodies against SORT1, ARHGAP33, GM130 (a Golgi marker) and EEA1 (an endosomal marker). (**c**) The amount of TrkB, but not SORT1, in the Golgi membrane-enriched fraction was significantly increased in *ARHGAP33* KO mice. Equal amount of the Golgi membrane fractions from WT and *ARHGAP33* KO mice were probed with anti-TrkB, anti-SORT1, anti-GM130 and anti-ARHGAP33 antibodies. (**d**) Quantification of the amount of TrkB and SORT1 in the Golgi membrane-enriched fraction (each, *n*=7, TrkB, *P*=0.0017; SORT1, *P*>0.05, Mann–Whitney *U*-test). The levels of TrkB and SORT1 in the Golgi-enriched fraction from *ARHGAP33* KO mice were normalized to those from WT mice (The averaged WT values were set to 100%). *P<0.05. NS, not significant. Bars show median values.

To elucidate the molecular function of ARHGAP33, we examined the subcellular localization of ARHGAP33 via immunofluorescent staining (Fig. 5a). In dissociated hippocampal neurons, ARHGAP33 was mainly located in the cell body, where it colocalized with the Golgi apparatus marker GM130 (Fig. 5a, upper). ARHGAP33 staining was not detected in neurons from *ARHGAP33* KO mice, indicating that the staining signal is specific to ARHGAP33 (Fig. 5a, lower). Our biochemical analysis showed that ARHGAP33 was most abundant in the Golgi membrane-enriched fraction (at the 0.25 M/1.1 M sucrose interface), as was evident from the enrichment of GM130 (Fig. 5b). These immunocytochemical and biochemical data indicate that ARHGAP33 localizes and may function at the Golgi apparatus. Interestingly, the amount of TrkB in the Golgi membrane-enriched fraction was significantly increased in *ARHGAP33* KO mice (*P*=0.0017; Fig. 5c,d), suggesting impaired TrkB trafficking at the Golgi apparatus. In contrast to TrkB, the amount of SORT1 in the Golgi membrane-enriched fraction was not significantly different between *ARHGAP33* KO and WT mice (Fig. 5c,d). Likewise, as previously shown in Fig. 1, the expression levels of SORT1 in the cell-surface fraction and in the PSD fraction were similar between *ARHGAP33* KO mice and WT mice. These results suggest that ARHGAP33 does not affect SORT1 localization in neurons.

### ARHGAP33 modulates the interaction of SORT1 with TrkB

**Figure 6:**
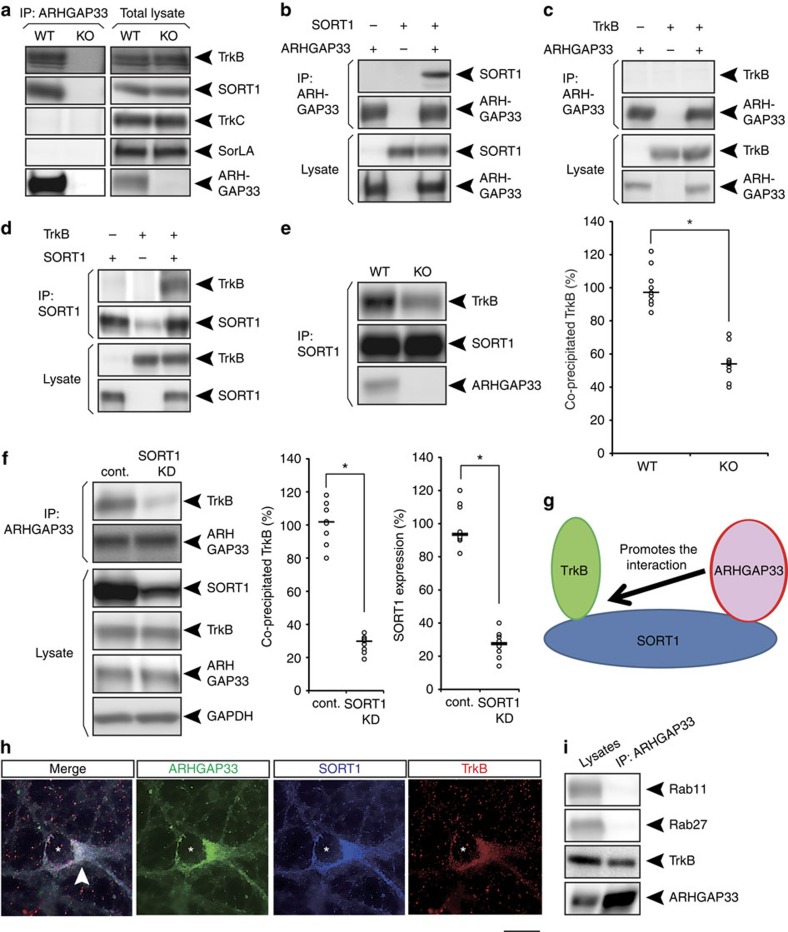
ARHGAP33 promotes the interaction between TrkB and SORT1. (**a**) ARHGAP33 formed complexes with SORT1 and TrkB. ARHGAP33-immunoprecipitates (left) and hippocampal total lysates (right) were immunoblotted with the indicated antibodies. (**b**–**d**) ARHGAP33 formed complexes with SORT1 and TrkB. HEK293T cells were transfected with ARHGAP33, SORT1 and TrkB, as indicated. Immunoprecipitates were immunoblotted with the indicated antibodies. (**e**) Weakened interaction between TrkB and SORT1 in *ARHGAP33* KO mice. SORT1 immunoprecipitates were immunoblotted with the indicated antibodies. Representative blots (left) and quantification of co-immunoprecipitated TrkB (right; each *n*=9; *P*=3.4 × 10^−4^, Mann–Whitney *U*-test). *P<0.05. The level of co-precipitated TrkB in *ARHGAP33* KO mice was normalized to that in WT mice (the averaged WT value was set to 100%). Bars show median values. Western blots show representative results from nine independent experiments performed using different mice. (**f**) Weakened interaction between ARHGAP33 and TrkB in the SORT1 knockdown neuron. ARHGAP33-immunoprecipitates and total lysates were immunoblotted with the indicated antibodies. Representative blots (left), quantification of co-immunoprecipitated TrkB (centre) and quantification of SORT1 expression (confirmation of SORT1 knockdown; right; each *n*=8; TrkB, *P*=7.5 × 10^−4^, SORT1, *P*=7.7 × 10^−4^, Mann–Whitney *U*-test). The averaged values of the control neurons were set to 100%. *P<0.05. Bars show median values. Western blots show representative results from eight independent experiments performed using neurons from different mice; cont., control; KD, knockdown. Note that the MISSION shRNA construct (TRCN0000034496) was used. (**g**) In the ARHGAP33/SORT1/TrkB complex, ARHGAP33 promoted the interaction between TrkB and SORT1. In addition, ARHGAP33 is suggested to recruit Cdc42 into the complex^7^. (**h**) Colocalization of ARHGAP33, SORT1 and TrkB at the perinuclear region. Triple immunostaining for ARHGAP33, SORT1 and TrkB in dissociated hippocampal neurons. Scale bar, 5 μm. The asterisks indicate the nucleus of the neuron. The arrowhead indicates the colocalization of these three proteins at the perinuclear region. The data are representative of five independent experiments. (**i**) Interactions between ARHGAP33 and Rab11 or Rab27 were not detected in the hippocampal lysates. ARHGAP33-immunoprecipitates (right) and hippocampal total lysates (left) were immunoblotted with the indicated antibodies.

The results showing increased TrkB at the Golgi apparatus in *ARHGAP33* KO mice led us to hypothesize that ARHGAP33 may interact with the Golgi-localized proteins that are involved in the intracellular trafficking of proteins^30^. One such protein is SORT1, a member of the Vps10 domain receptor family that regulates the sorting of TrkB at the Golgi apparatus^19^,^20^. We confirmed the result of a previous report that SORT1 is most abundant in the Golgi membrane-enriched fraction in the 0.25 M/1.1 M sucrose interface (Fig. 5b)^17^. Using hippocampal brain lysates from *ARHGAP33* KO and WT mice, we found that ARHGAP33 is associated with SORT1 (Fig. 6a). SorLA^31^, another brain-enriched Vps10 domain receptor family member, did not co-immunoprecipitate with ARHGAP33, demonstrating selective binding between ARHGAP33 and SORT1 (Fig. 6a). Interestingly, as in the case of SORT1, TrkB co-immunoprecipitated with ARHGAP33 (Fig. 6a), suggesting that the ARHGAP33–SORT1–TrkB complex may be present in the brain. In contrast to TrkB, TrkC did not co-immunoprecipitate with ARHGAP33 (Fig. 6a). We also confirmed the ARHGAP33–SORT1–TrkB interaction after transfection of plasmids encoding these proteins into HEK293T cells in which the endogenous expression of these proteins was hardly detectable (Fig. 6b–d). We found that ARHGAP33 interacted with SORT1 (Fig. 6b), ARHGAP33 did not interact with TrkB in the absence of SORT1 (Fig. 6c) and SORT1 interacted with TrkB (Fig. 6d). These results suggest that ARHGAP33 interacts directly with SORT1 but not with TrkB. Interestingly, using hippocampal brain lysates, we determined that the interaction between SORT1 and TrkB was reduced in the absence of ARHGAP33 (*P*=3.4 × 10^−4^; Fig. 6e). These data suggest that ARHGAP33 may act as a scaffolding protein that facilitates the formation of SORT1-containing trafficking complexes, where ARHGAP33 is likely to promote the interaction of SORT1 with TrkB. The precise mechanism that the interaction between SORT1 and TrkB requires ARHGAP33 remains unclear. One possibility is that ARHGAP33 may induce conformational changes in SORT1. Further studies are needed to elucidate the mechanism. Furthermore, we performed SORT1 knockdown by two non-overlapping shRNAs in WT neurons (Fig. 6f, Supplementary Fig. 4). An shRNA sequence that does not target any mouse genes (SHC002, Sigma-Aldrich) was used as control. We found that the interaction of ARHGAP33 with TrkB was significantly weakened in the SORT1-knockdown neurons (*P*=7.5 × 10^−4^; Fig. 6f, Supplementary Fig. 4), suggesting that SORT1 is essential for the formation of ARHGAP33/TrkB/SORT1 complexes (Fig. 6g). We then examined the colocalization of ARHGAP33, SORT1 and TrkB via immunofluorescent staining. In dissociated hippocampal neurons, these proteins were colocalized mainly at the perinuclear region, presumably in the Golgi apparatus (Fig. 6h). In addition, previous studies have reported that TrkB trafficking is mediated by small GTPases of the Rab family, such as Rab11 and Rab27 (refs 32, 33). We did not detect these Rab proteins in the ARHGAP33 complex (Fig. 6i), suggesting that ARHGAP33 may not be involved in Rab-mediated trafficking.

### ARHGAP33 and SORT1 cooperates to facilitate TrkB trafficking

**Figure 7:**
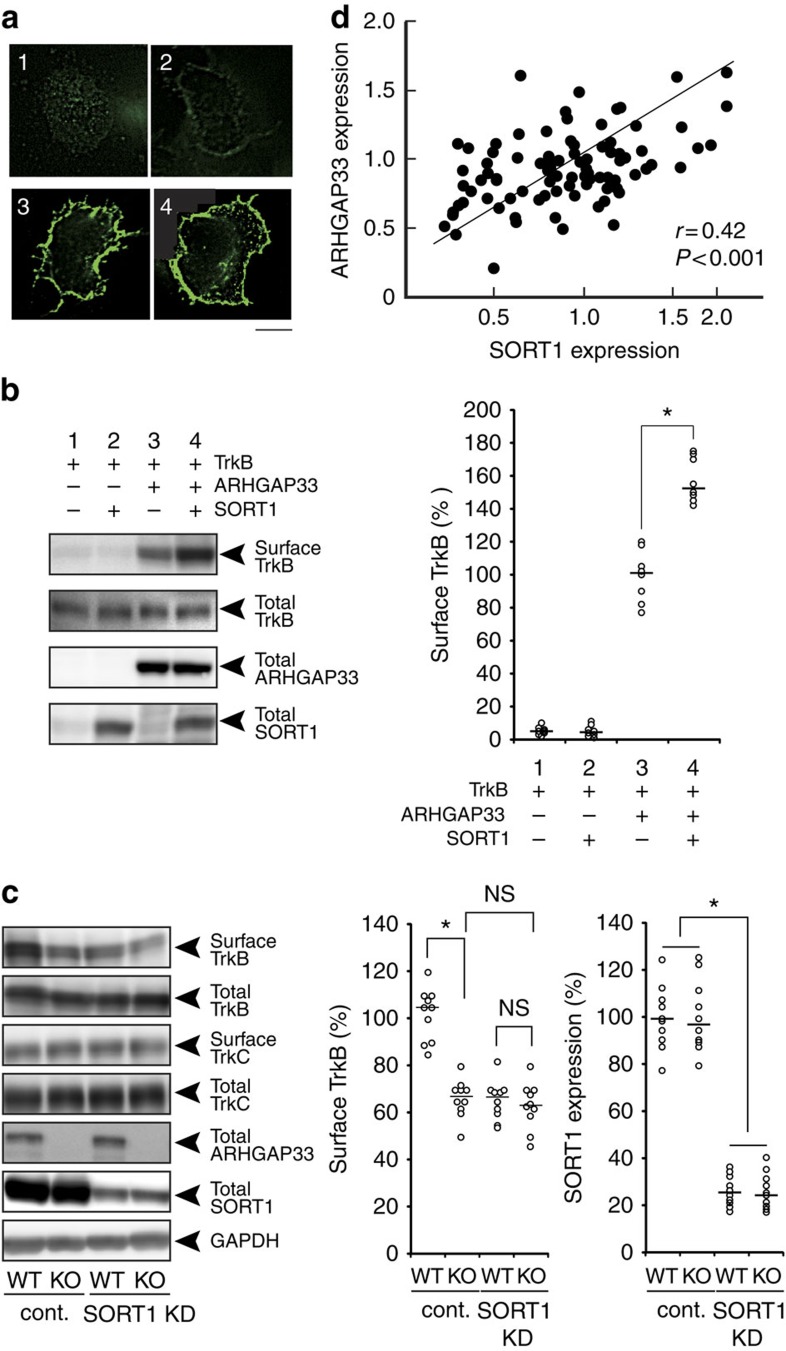
Cooperative facilitation of TrkB trafficking by ARHGAP33 and SORT1. (**a**,**b**) Increased TrkB surface expression by ARHGAP33 and SORT1. HEK293T cells were transfected with TrkB, ARHGAP33 and SORT1 as indicated in **b** (1–4). Representative data from eight independent experiments of immunostaining of surface TrkB in HEK293T cells with an antibody against the extracellular region of TrkB (**a**). Scale bar, 10 μm. Representative blots (left) and the quantification of the surface TrkB (right) (**b**). Biotinylated cell-surface proteins were immunoblotted with the indicated antibodies. The surface expression of TrkB was enhanced by simultaneous expression of ARHGAP33 and SORT1 compared with the expression of ARHGAP33 alone (each *n*=8; ARHGAP33 alone versus ARHGAP33 and SORT1, *P*=0.004, Kruskal–Wallis test followed by *post hoc* Steel–Dwass tests). Western blots show representative results from eight independent experiments. The averaged value of surface TrkB level in cells expressing ARHGAP33 alone (lane 3) was set to 100%. *P<0.05. Bars show median values. (**c**) Requirement of SORT1 in ARHGAP33-mediated TrkB trafficking. Biotinylated cell-surface proteins were immunoblotted with the indicated antibodies. Representative blots (left), quantification of surface TrkB expression (centre) and quantification of SORT1 expression (confirmation of SORT1 knockdown; right; each *n*=10; surface TrkB, WT versus *ARHGAP33* KO in control neurons, corrected *P*=6.0 × 10^−4^; WT versus *ARHGAP33* KO in SORT1 knockdown neurons, corrected *P*>0.05; *ARHGAP33* KO versus *ARHGAP33* KO plus SORT1 knockdown, corrected *P*>0.05, Mann–Whitney *U*-test with the Ryan's correction). Western blots show representative results from 10 independent experiments performed using neurons from different mice. The averaged values of WT mice in the control neurons were set to 100%. *P<0.05; cont., control; KD, knockdown; NS, not significant. Bars show median values. Note that the MISSION shRNA construct (TRCN0000034496) was used. (**d**) Strongly correlated expression of *SORT1* and *ARHGAP33* in immortalized lymphocytes from human blood (r=0.42, *P*<0.001, Spearman's rank order correlation test). Quantitative RT-PCR analysis of *ARHGAP33* and *SORT1* expression in immortalized lymphocytes was performed. Then, the levels of *SORT1* and *ARHGAP33* expression in each sample were plotted.

We then examined the functional interaction of ARHGAP33 with SORT1 in TrkB transport. As reported previously, the expression of SORT1 alone failed to increase TrkB cell-surface expression in HEK293T cells (Fig. 7a,b, 1 versus 2)^19^. In contrast, exogenous expression of ARHGAP33 increased TrkB cell-surface expression without affecting the total TrkB levels in HEK293T cells (Fig. 7a,b, 1 versus 3). This result is consistent with the decreased cell-surface expression of neuronal TrkB in *ARHGAP33* KO mice (Fig. 1b,c). Interestingly, the simultaneous expression of ARHGAP33 and SORT1, as compared with the single expression of ARHGAP33, increased the cell-surface expression of TrkB to significantly higher levels (*P*=0.004; Fig. 7a,b, 3 versus 4). We then examined the role of SORT1 in ARHGAP33-mediated trafficking of TrkB by comparing the effects of SORT1 knockdown between WT and *ARHGAP33* KO mice (Fig. 7c, Supplementary Fig. 4). In contrast to the clear difference between the two mouse strains in control neurons infected with an shRNA that had no target mouse genes, *ARHGAP33* deficiency virtually had no additive effect on the surface expression of TrkB in SORT1 knockdown neurons (Fig. 7c, Supplementary Fig. 4; WT versus *ARHGAP33* KO in control neurons, corrected *P*=6.0 × 10^−4^; WT versus *ARHGAP33* KO in SORT1 knockdown neurons, corrected *P*>0.05; *ARHGAP33* KO versus *ARHGAP33* KO plus SORT1 knockdown, corrected *P*>0.05). These data suggest that ARHGAP33 and SORT1 function cooperatively to increase TrkB trafficking to the cell surface and that, in addition to SORT1, ARHGAP33 may interact with other protein(s) to regulate TrkB trafficking.

### Association of *ARHGAP33* genetic variants with schizophrenia

**Figure 8:**
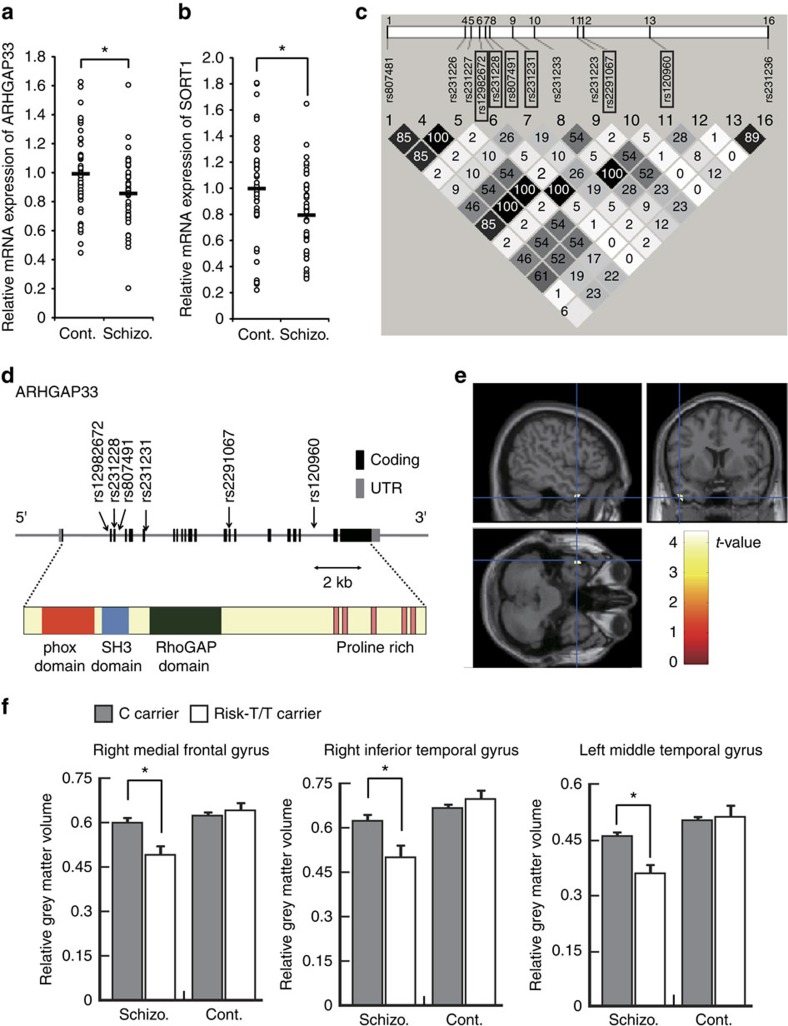
Association of *ARHGAP33* with schizophrenia. (**a**,**b**) Quantitative RT–PCR analysis of *ARHGAP33* and *SORT1* expression in immortalized lymphocytes from schizophrenia patients (schizo.) and age- and sex-matched controls. Significant reductions are observed in *ARHGAP33* (each *n*=45, *U*=699, *P*=0.011, Mann–Whitney *U*-test; **a**) and *SORT1* (each *n*=45; *U*=683, *P*=7.8 × 10^−3^, Mann–Whitney *U*-test; **b**) in schizophrenia patients. The expression levels of these genes were normalized to *GAPDH* mRNA. Bars show median values. (**c**) Linkage disequilibrium of *ARHGAP33* in the HapMap JPT. Each diamond represents the correlation (*r*^2^) between each pair of SNPs, with darker shades representing stronger linkage disequilibrium, as obtained from the HapMap JST samples. (**d**) The locations of the SNPs analysed in this study. (**e**,**f**) Impact of the risk-T-allele on grey matter volume of the left middle temporal gyrus in schizophrenia patients. A significant cluster of the genotype effect in the left middle temporal gyrus is observed in schizophrenia patients, which is shown as cross-hairline (uncorrected *P*<0.001, cluster size >100; **e**). Relative grey matter volumes extracted from the left middle temporal gyrus (F_1,122_=13.5, *P*=3.6 × 10^−4^, ANOVA), the right medial frontal gyrus (F_1,122_=13.5, *P*=8.2 × 10^−4^, ANOVA) and the right inferior temporal gyrus (F_1,122_=13.1, *P*=4.4 × 10^−4^, ANOVA; **f**). *P<0.05. Data are expressed as the mean±s.e.m.

Our results that *ARHGAP33* KO mice exhibited neuropsychiatric disorder-like abnormal behaviour (Fig. 3) suggest that human *ARHGAP33* might be associated with schizophrenia or some other neuropsychiatric disorders. To test this possibility, we first examined whether the levels of *ARHGAP33* and *SORT1* expression are correlated in human tissues. In immortalized lymphocytes from human blood samples (Supplementary Table 1), we observed a highly significant correlation between *ARHGAP33* and *SORT1* expression levels (Fig. 7d; *r*=0.42, *P*<0.001), suggesting that common cellular mechanisms regulate the expression of *ARHGAP33* and *SORT1*. Interestingly, the expression levels of *ARHGAP33* and *SORT1* normalized to the expression of *GAPDH* were significantly lower in the immortalized lymphocytes from 45 patients with schizophrenia compared with those from 45 age- and sex-matched controls (Fig. 8a,b; *ARHGAP33*, *P*=0.011*; SORT1*, *P*=7.8 × 10^−3^; an analysis of covariance with sex and age as covariates did not alter the results (*ARHGAP33*, F=7.2, *P*=8.8 × 10^−3^; *SORT1*, F=7.8, *P*=6.5 × 10^−3^)). These results suggest that ARHGAP33 and its functional interactor, SORT1, may be involved in the pathophysiology of neuropsychiatric disorders. Then, we examined the possible association between schizophrenia and genetic variation in the *ARHGAP33* gene by genotyping six tagging single-nucleotide polymorphisms (SNPs) located in the *ARHGAP33* gene and its flanking regions (Fig. 8c,d). Significant differences in the allelic frequencies of rs12982672 and rs231228 were observed between patients and controls (rs12982672, uncorrected *P*=0.021; rs231228, uncorrected *P*=0.0064; Table 1). The difference remained significant for rs231228 but not for rs12982672 after the Bonferroni correction for multiple SNP tests (rs231228, corrected *P*=0.038; rs12982672, corrected *P*=0.13). The frequency of the minor T allele of rs231228 was higher in patients (0.26) than in controls (0.23; odds ratio (95% confidence interval)=1.14 (1.04–1.26); Table 1). These data suggest that the *ARHGAP33* gene is genetically associated with schizophrenia. In addition, there was no allelic association of any of the other SNPs with schizophrenia (uncorrected *P*>0.05; Table 1).

### Effect of the *ARHGAP33* polymorphism on brain structure

We next examined the possible impact of the SNP rs231228 of the *ARHGAP33* gene on the neurobiological traits. We performed an exploratory whole-brain analysis to investigate the risk association with rs231228 genotype and the genotype–diagnosis interaction in grey matter volumes for 124 schizophrenia patients and 407 controls (Supplementary Table 2). We observed significant effects of the genotype–diagnosis interaction on the left middle temporal gyrus (corrected *P*=7.2 × 10^−4^), the right medial frontal gyrus (corrected *P*=1.1 × 10^−3^), and the right inferior temporal gyrus (corrected *P*=8.5 × 10^−3^; T>3.69; Fig. 8e,f and Supplementary Table 3). We did not observe a genotype effect among the subjects (uncorrected *P*>1.0 × 10^−3^). In schizophrenia patients, the grey matter volume in the left middle temporal gyrus was smaller in the subjects harbouring risk homozygous T/T allele of rs231228 than in the subjects harbouring C/C or C/T alleles of rs231228 (*P*=3.6 × 10^−4^, Fig. 8f). Likewise, grey matter volumes in the other two regions were smaller in the risk homozygous T patients than in the C carriers (right medial frontal gyrus, *P*=8.2 × 10^−4^; right inferior temporal gyrus, *P*=4.4 × 10^−4^; Fig. 8f). However, in the controls, there was no significant difference in grey matter volumes between the risk homozygous T subjects and C carriers (Fig. 8f; *P*>0.05). These data suggest that the *ARHGAP33* polymorphism may be associated with several morphological brain vulnerabilities in schizophrenia patients.

## Discussion

In this study, we discover a unique role for ARHGAP33 as a brain-enriched SNX protein in the intracellular trafficking of TrkB. *ARHGAP33* KO mice exhibited impaired spine morphogenesis and neuropsychiatric disorder-related behavioural abnormalities. Importantly, human *ARHGAP33* was associated with the brain phenotypes exhibited by schizophrenia patients (Fig. 8). Furthermore, a correlated decrease in the *ARHGAP33* and *SORT1* expression levels was observed in the peripheral lymphocytes of schizophrenia patients (Fig. 7d). Taken together, our results suggest that ARHGAP33-mediated intracellular trafficking of TrkB is crucial for synapse development and that its dysfunction might be related to the pathophysiology of neuropsychiatric disorders.

TrkB is produced in the cell body and transported to synaptic sites via anterograde transport, but the mechanism of that transport is not well understood^34^. In addition, the possible involvement of SNX proteins in TrkB trafficking has not been investigated. This study revealed that an ARHGAP33 deficiency resulted in the accumulation of TrkB in the Golgi apparatus (Fig. 5c,d). Because ARHGAP33 is enriched in the Golgi apparatus, this SNX protein is expected to be involved in TrkB trafficking at the Golgi apparatus. Interestingly, SORT1 and Cdc42, a substrate for ARHGAP33, localize primarily at the Golgi apparatus and are involved in the exit of proteins from the Golgi apparatus^19^,^20^,^35^. Thus, ARHGAP33 may act as a scaffolding protein that facilitates the formation of Cdc42/SORT1-containing trafficking complexes to enhance TrkB exit from the Golgi apparatus (Fig. 6g). Although SORT1 has been shown to facilitate the intracellular sorting of BDNF into the regulated secretion pathway^17^, BDNF secretion into the medium was normal in the dissociated hippocampal neurons from *ARHGAP33* KO mice (WT, 100±10.2%; *ARHGAP33* KO, 95±9.5%; *P*>0.05, Mann–Whitney *U*-test). We also found normal surface expression of another Trk family member, TrkC, in *ARHGAP33* KO mice (Fig. 1b,c), indicating the selective nature of the ARHGAP33-mediated regulation of protein transport.

We previously identified another ARHGAP33 family protein, ARHGAP32, that regulates spine morphogenesis^11^,^12^. These two proteins share a common multi-domain architecture composed of an amino (N)-terminal PX domain, an SH3 domain and a RhoGAP domain^4^. ARHGAP32 has been reported to mediate endoplasmic reticulum to-Golgi transport of the N-cadherin/β-catenin complex in fibroblasts^10^, although its functional significance in the brain remains to be clarified. Interestingly, we recently reported that the *ARHGAP32* gene is associated with an increased risk of schizophrenia and schizotypal personality traits^36^. Consistent with this previous finding, we discovered for the first time that human *ARHGAP33* is associated with schizophrenia in the present study. We also revealed that *ARHGAP33* KO mice exhibited a clear impairment in PPI, working memory and habituation (Fig. 3), which are symptoms that are often observed in both mouse models and human schizophrenia patients^24^,^25^. Taken together, our present results suggest that both ARHGAP32 and ARHGAP33 may be involved in the pathophysiology of schizophrenia. Importantly, SORT1, a regulator of SNX-mediated intracellular protein trafficking, may also be involved in schizophrenia (Fig. 8)^17^. Previous studies have identified a number of susceptibility genes for schizophrenia, many of which regulate synapse formation, function and plasticity^18^,^37^,^38^,^39^,^40^. In this respect, ARHGAP33 and ARHGAP32 as well as SORT1 are unique in that they are regulators of intracellular protein trafficking, highlighting a novel role of the SNX-related proteins in the pathophysiology of schizophrenia. However, several important points related to this idea remain unclear, including the biological significance of the identified genetic variation of *ARHGAP33*.

Previous studies have found that the impairment of multiple processes related to TrkB regulation at the stages of transcription, translation and post-translational regulation are involved in schizophrenia (for review, see ref. 16). For example, post-mortem studies have revealed that both mRNA and protein levels of BDNF and TrkB are decreased in multiple cortical areas in schizophrenia patients^41^. A truncated TrkB lacking its kinase domain, which acts as a dominant-negative receptor for BDNF and prevents TrkB/BDNF signalling, has been reported to be increased in schizophrenia patients^42^. However, there has been no report thus far regarding the involvement of TrkB trafficking in schizophrenia. Our current results suggest that impaired ARHGAP33-mediated TrkB trafficking is involved in neuropsychiatric disorder-related deficits in mice (Figs 2 and 3), although further studies are needed to establish convincing links among the ARHGAP33-mediated TrkB trafficking, mature brain function and neuropsychiatric disorders. In addition to ARHGAP32 and ARHGAP33, other SNX proteins are involved in brain functions. For example, SNX27 has been reported to regulate excitatory synaptic functions by regulating glutamate receptor trafficking and is involved in Down's syndrome^43^,^44^,^45^. It will be interesting to determine whether ARHGAP33, ARHGAP32 or other SNX proteins function differentially or in coordination in neurons.

The odds ratio for the allelic variant of *ARHGAP33* is small (1.14; Table 1). This may be ascribed to polygenic inheritance in schizophrenia. Schizophrenia is likely to be caused by variations in multiple genes, each with a small impact on disease risk. The general trend across many studies is that the schizophrenia-associated SNPs have small effects on the disease risk, with odds ratios generally ranging from 1.05 to 1.2 (for review, see ref. 46). Thus, it seems reasonable that the odds ratio for the SNP rs231228 is small (1.14). The SNP rs231228 might require additional mutations to result in the disease phenotype. Future work is needed to determine the precise role of ARHGAP33 in the pathophysiology of schizophrenia.

The most significant SNP of *ARHGAP33* is localized in exon 3 and the cortex volume is reduced in the T/T carriers (Fig. 8). The exon 3 of *ARHGAP33* encodes the N-terminal region just upstream of the PX domain of ARHGAP33 in both human and mouse. We did not find any conserved functional domains in the region. Therefore, the exact molecular mechanism for reduced brain volume in the risk T/T carriers is currently unknown. The decreased expression of *ARHGAP33* in patients with schizophrenia (Fig. 8) may underlie the reduced brain volume. We found substantial changes in grey matter volume associated with the *ARHGAP33* risk allele while complete absence of ARHGAP33 in the *ARHGAP33* KO mice had no apparent effect on the brain structure. This discrepancy may be caused by methodological differences in the imaging experiments between human subjects and mice. Generally, technologies for whole-brain imaging in human subjects are much further along than those in rodents: in this study, magnetic resonance imaging (MRI) images of human subjects were analysed using optimized voxel-based morphometry method, followed by segmenting the grey matter from spatially normalized images and smoothing the grey-matter segments for unbiased anatomical comparison across the whole brain. With these well-established technologies, we can detect slight abnormalities in brain volume in human subjects (Fig. 8). Currently, it is very difficult to apply the similar technology for the analysis of mouse brain because the brain size is too small for the voxel-based morphometry method. We might be able to find an abnormality in the brain structure of *ARHGAP33* KO mice in future, if new technologies for whole-brain imaging are developed in mice. Alternatively, abnormalities in mouse brains can be assessed post-mortem by examining the brain sections or by weighing the brain regions.

Although previous studies have reported that ARHGAP/NOMA-GAP-KO mice exhibit decreased cortical thicknesses associated with oversimplification of dendritic arborization^8^, we did not detect these abnormalities in our KO mice (Supplementary Fig. 2). This discrepancy may be explained by several factors, such as a difference in targeted deletion region, LacZ insertion and strain difference. However, we report for the first time that ARHGAP33 regulates higher brain functions such as learning and memory and is likely involved in neuropsychiatric disorders.

In conclusion, we identified ARHGAP33 as a new type of regulator for intracellular protein trafficking. Given that the neuropsychiatric disorder-related deficits in *ARHGAP33* KO mice could be reversed even in adulthood, ARHGAP33-mediated TrkB trafficking may be a unique target for pharmacological intervention. The adult *ARHGAP33* KO mouse is an animal model of neuropsychiatric disorders in which compounds that target TrkB-mediated signalling can be evaluated.

## Methods

### Ethical statement and animal experiments

This study was performed in accordance with the World Medical Association's Declaration of Helsinki and was approved by the Research Ethical Committee of Osaka University, Fujita Health University, Nagoya University, Tokushima University, Juntendo University and The University of Tokyo. Animal experiments were performed in accordance with the guidelines for animal use issued by the Committee of Animal Experiments, The University of Tokyo, and approved by the Committee.

### Generation of *ARHGAP33* KO mice

*ARHGAP33* KO mice were generated from E14.1 ES cells (Macrogen Inc., Seoul, Korea; Supplementary Fig. 2). Heterozygous *ARHGAP33* KO mice were successively backcrossed to C57BL/6J mice to yield subsequent generations with an almost pure C57BL/6J genetic background. F10 heterozygous mice were crossed to each other to yield homozygous mice and WT littermates. This study used 12–18-week-old male mice. Animals were housed in a 12/12 h light/dark cycle, in litters of four to eight before weaning (P28) and three to six per cage after weaning. Animal experiments were performed in accordance with the guidelines for animal use issued by the Committee of Animal Experiments, The University of Tokyo, and approved by the Committee.

### Fluorescence *in situ* hybridization

Complementary DNA fragments encoding mouse *ARHGAP33* cDNA (GenBank accession number, XM_003467216) was used for generating cRNA probe. Simultaneous detection of multiple mRNAs via fluorescence *in situ* hybridization using digoxigenin-labelled cRNA probes was performed as described previously^47^. No randomization or blinding was used in any biochemical analysis. All recombinant DNA experiments were reviewed and approved by our institutional committees.

### Antibodies

To generate a monoclonal antibody against ARHGAP33, a fragment of mouse ARHGAP33 was used as an immunogen (UNITECH, Kashiwa, Japan). Rabbit polyclonal anti-TrkB antibodies were generated using an extracellular domain of mouse TrkB (unpublished). The rabbit polyclonal anti-ARHGAP33 antibodies were described previously^7^. Commercially available antibodies used were as follows: anti-ARHGAP33 (#HPA030118) antibody (Atlas Antibodies, Stockholm, Sweden), anti-GM130 (#610822) and anti-EEA1 (#9001964) antibodies (BD Transduction Laboratories, CA, USA), anti-PSD95 antibody (#MA1–046; Affinity Bioreagents, CO, USA), anti-TrkC (#3376) and anti-Tubulin (#2125) antibodies (Cell Signaling, MA, USA), anti-SORT1 (#ab16640) and anti-TrkB (#ab89925) antibodies (Abcam, Cambridge, UK), anti-SorLA (#sc136073) antibody (SantaCruz, CA, USA), anti-GM130 antibody (#G7295; Sigma, MO, USA), anti-phosphotyrosine (4G10) antibody (#05–1050; Millipore, MA, USA), anti-SORT1 antibody (#ANT-009; Alomone labs, Jerusalem, Israel), anti-Rab11 antibody (#71–5300; Invitrogen, MA, USA) and anti-Rab27 antibody (#18975; IBL, Gunma, Japan). All the antibodies were used at concentrations of 0.5–1 μg ml^−1^.

### Immunoblotting

Lysates of HEK293T cells, cultured hippocampal neurons and hippocampi isolated from mouse brains were prepared using standard protocols, as described previously^11^. Briefly, HEK293T cells, cultured hippocampal neurons and hippocampi were lysed with TNE buffer. For immunoprecipitation, lysates were cleared by centrifugation with an excess amount of protein G-sepharose and then incubated with indicated antibodies on ice for 1 h. Immune complexes were collected on protein G-sepharose and washed five times with TNE buffer. Immunoprecipitates or lysates were resolved by SDS–PAGE (polyacrylamide gel electrophoresis) and transferred to PVDF membranes. Then the membranes were probed with antibodies indicated. Data acquisition and analysis were performed using LAS 4000 (GE Healthcare, NJ, USA). Data were statistically analysed with Mann-Whitney *U*-test with the Ryan's correction as necessary for the individual experiments, or Kruskal–Wallis test followed by Steel–Dwass tests. Differences were considered significant at *P*<0.05. For details, see figure legends.

### Nissl staining

Under deep pentobarbital anesthesia, mice were transcardially perfused with 4% paraformaldehyde in PBS. The brains were removed, frozen and sectioned at a thickness of 16 μm using a CM3050S cryostat. After wetting with PBS and distilled water, the sections were stained with 0.5% cresyl violet/0.01% acetic acid. The sections were then rinsed with distilled water. The sections were photographed using a BZ-9000 microscope (Keyence, Osaka, Japan).

### Hippocampal neuronal cultures and immunocytochemistry of neurons

Hippocampal cultures were prepared from E16.5 embryonic mouse hippocampi in MEM with B27 supplement and 5% FBS and plated on glass coverslips coated with poly-L-lysine, as described^11^. Two days after plating, 10 μM ara-C was added to prevent glial cell proliferation. Immunocytochemistry of the neurons was performed as described previously^11^. For labelling antibodies, Zenon Rabbit IgG labeling kit was used according to the instructions (Molecular Probes, OR, USA).

### Surface biotinylation assay

The receptor biotinylation assays were performed using a cleavable biotin, as described previously^48^. The surface proteins of hippocampal cultures at 14 days *in vitro* and HEK293T cells were biotinylated with 1 mg ml^−1^ sulfo-NHS-SS-biotin (Pierce, IL, USA) for 20 min at 4 °C. To collect the surface proteins, cells were lysed with lysis buffer (20 mM HEPES (pH 7.5), 100 mM NaCl, 1 mM EGTA, 1% NP-40, 1% sodium deoxycholate, 0.01% SDS) and biotinylated proteins were precipitated with NeutrAvidin resins (Pierce).

### Preparation of PSD fraction

The preparation of the PSD fraction was performed as described previously^12^. Briefly, mouse brains were homogenized in ice-cold HEPES buffer using a Dounce homogenizer. The supernatant of the brain homogenate centrifuged at 1,000*g* was again centrifuged at 12,500*g* to obtain a crude synaptosomal fraction (P2). The P2 fraction was lysed hypo-osmotically and centrifuged again to pellet the synaptosomal membrane fraction. The synaptosomal membrane fraction was treated with a HEPES-based buffer containing 1% Triton X-100, and the Triton X-100 insoluble fraction was obtained by centrifugation. Purified PSD fraction, which was finally obtained by centrifugation of the Triton X-100 insoluble fraction with a density gradient, was dissolved by 1% SDS buffer.

### Golgi impregnation and dendritic spine analysis

Golgi impregnation was performed on 14-week-old WT and *ARHGAP33* KO mice with the FD rapid GolgiStain Kit (FD Neuro Technologies, MD, USA), according to the manufacturer's protocol. Fully focused images were obtained by automatically merging focused areas of z-stack images using a BZ-9000 microscope and the bundled analysis software (Keyence). To examine the effect of 7,8-DHF on spine morphology, mice were intraperitoneally administered 7,8-DHF (12.5 mg kg^−1^; Tokyo Chemical Industry Co. Ltd., Tokyo, Japan) once per day for 14 days starting at 12 weeks of age. We performed a blind analysis of the images using ImageJ software. Dendritic protrusions with widths larger than half their length were classified as mature spines^49^. The data were statistically analysed using one-way analysis of variance (ANOVA) or two-way ANOVA followed by the Tukey-Kramer *post hoc* test. Differences were considered significant at *P*<0.05.

### Electrophysiology

All electrophysiological experiments were blindly performed at 25–28 °C. Coronal hippocampal slices (400-μm thick) were prepared from 12- to 14-week-old male WT or *ARHGAP33* KO littermates for the measurements of the paired-pulse ratio of evoked EPSCs and the amplitude and frequency of mEPSCs, as previously described^50^,^51^. In brief, mice were decapitated under anesthesia with 100% CO_2_, and the brains were cooled in an ice-cold modified external solution (120 mM Choline-Cl, 2 mM KCl, 8 mM MgCl_2_, 28 mM NaHCO_3_, 1.25 mM NaHPO_4_ and 20 mM glucose (bubbled with 95% O_2_ and 5% CO_2_)). Slices were cut using a Leica VT1200 slicer (Leica Microsystems). For recovery, slices were incubated for at least 1 h in a normal bath solution (125 mM NaCl, 2.5 mM KCl, 2 mM CaCl_2_, 1 mM MgSO_4_, 1.25 mM NaH_2_PO_4_, 26 mM NaHCO_3_ and 20 mM glucose (pH 7.4; bubbling with 95% O_2_ and 5% CO_2_)). The recording chamber was perfused with the external solution supplemented with 100 μM picrotoxin (Tocris, Bristol, UK) for recording evoked EPSCs. TTX (0.5 μM; Nacalai, Kyoto, Japan) and an NMDA receptor antagonist (R)-CPP (10 μM; Tocris) were added to the picrotoxin-containing external solution for recording mEPSCs.

Whole-cell recordings were made from dentate gyrus granule cells in the hippocampus using an upright microscope (BX50WI, Olympus) equipped with an infra-red CCD camera system (Hamamatsu Photonics K.K., Hamamatsu, Japan)^52^. Resistance of the patch pipette was 2–3 MΩ when filled with the intracellular solution of the following composition: 140 mM CsCl, 10 mM HEPES, 10 mM BAPTA-K_4_, 4.6 mM MgCl_2_, 4 mM Na_2_-ATP and 0.4 mM Na_2_-GTP (pH 7.3, adjusted with CsOH). Membrane currents were recorded using an EPC9/2 amplifier (HEKA Electronik, Lambrecht/Pfalz, Germany) and the pipette access resistance was compensated by 80%. The PULSE software (HEKA Electronik) was used for stimulation and data acquisition. The Mini analysis program (Ver. 6.0.3, Synaptosoft Inc., GA, USA) was used for analysing the mEPSC data. The signals were filtered at 3 kHz and digitized at 40 kHz. For synaptic stimulation, two glass micropipettes filled with normal saline were placed in the middle third of the molecular layer. Stimulus pulses (duration: 0.1 ms; intensity: 0–80 V) were applied between the pipettes to evoke EPSCs in granule cells. The paired-pulse ratio was determined at a holding potential of −70 mV as the ratio of the second to the first peak amplitude of AMPA receptor-mediated EPSCs at an interstimulus interval of 50 ms in the normal bathing solution supplemented with 100 μM picrotoxin (Tocris). All data were included in the analysis. Data were statistically analysed using the Mann–Whitney *U*-test and the Kolmogorov–Smirnov test. Differences were considered significant at *P*<0.05.

### Behavioural analysis

In each behavioural experiment, 12–18-week-old male WT or *ARHGAP33* KO littermates (F10, C57BL/6J genetic background) were analysed. Independent groups of mice were used for each experiment except for the open-field test, the contextual fear-conditioning test, the Morris water maze test and the elevated plus maze test. No statistical method was used to predetermine the sample size. All behavioural experiments were performed blindly during the light period. No randomization was used. All data were included in the analysis.

The Y-maze was composed of three equally spaced troughs radiating from a triangular central area (O'Hara & Co. Ltd., Tokyo, Japan). Each arm was 30 cm long, 16 cm high, 3 cm wide at the bottom and 10 cm wide at the top. Each mouse was placed at the end of one arm facing the centre and allowed to freely explore the apparatus with the experimenter out of sight. All sessions were video-recorded via a camera mounted above the maze and behaviour was evaluated using Time YM1 software (O'Hara & Co. Ltd.). Alternation behaviour was defined as consecutive entries into each of the three arms without repetition. We defined the percentage of spontaneous alternations as the actual alternations divided by the possible alternations (total entries−2) × 100. To examine the effect of 7,8-DHF on spatial working memory, mice were intraperitoneally administered 7,8-DHF (12.5 mg kg^−1^; Tokyo Chemical Industry Co. Ltd.) for 2 weeks. On the test day, mice were intraperitoneally administered 7,8-DHF 30 min before the test. The data were statistically analysed using either one-way ANOVA or two-way ANOVA for genotype, drug treatment and genotype × drug treatment interaction followed by Tukey–Kramer *post hoc* tests. Differences were considered significant at *P*<0.05.

A startle reflex measurement system (Mouse Startle; O'Hara & Co. Ltd.) was used for assessing the acoustic startle response and PPI. Before the tests, mice were exposed to restraint stress in a tube for 1 h per day for 13 days. To test the startle response, each mouse was placed into a small cylinder (30 or 35 mm in diameter, 12 cm long). The cylinder was placed on a sensor block in a soundproof chamber [60 × 50 × 67 cm (H)], and 65-dB white noise was presented as background noise. Mice were acclimatized to this experimental condition for 5 min, and then the experimental session was performed as described previously^53^. The following formula was used to calculate the percentage of PPI of the startle response: 100−[100 × (startle response on pre-pulse trials/startle response on 120 dB startle trials)]. The data were statistically analysed using the Friedman test followed by Scheffe tests. Differences were considered significant at corrected *P*<0.05.

Spontaneous locomotor activity was quantified in an open-field apparatus [30 × 30 × 40 cm (H)]. Briefly, each subject was placed in the centre of the open field apparatus and allowed to move freely for 10 min. The total distance travelled in the arena was recorded and analysed using Image OFCR 1.00 × and Image OF circle 1.01 × (O'Hara & Co. Ltd). This procedure was repeated daily for 4 days. To examine the effect of 7,8-DHF on spatial working memory, mice were intraperitoneally administered 7,8-DHF (12.5 mg kg^−1^; Tokyo Chemical Industry Co. Ltd.) for 2 weeks. On the test day, mice were intraperitoneally administered 7,8-DHF 30 min before the test. The data were statistically analysed using a repeated two-way ANOVA for genotype followed by Tukey–Kramer *post hoc* tests or a non-parametric Mann–Whitney *U*-test with the Ryan's correction. Differences were considered significant at *P*<0.05.

The accelerated rotarod test was performed as previously described^48^. The data were statistically analysed using two-way ANOVA with repeated measures for genotype. Differences were considered significant at *P*<0.05.

The open-field test was performed as previously described^48^. The data were statistically analysed using two-way ANOVA with repeated measures for genotype. Differences were considered significant at *P*<0.05.

Fear conditioning was conducted in a small conditioning chamber surrounded by a sound-attenuating chest (CL-M3, O'Hara & Co., Ltd., Tokyo, Japan). On day 1, mice were placed in the conditioning chamber for 10 s and presented with a tone of 65 dB/10 kHz for 10 s through a speaker on the roof of the chest. At the end of the tone presentation, a footshock (2 s/0.35 mA) was paired with the tone. Freezing responses were monitored for 1 min after the footshock, and the mice were then returned to their home cages. On day 2, the mice were placed in the conditioning chamber and freezing was scored for 6 min. Freezing responses were analysed with Image FZC 2.22sr2 software (O'Hara & Co., Ltd.), which is software based on the NIH Image program. The data were statistically analysed using two-way ANOVA with repeated measures for genotype (with time as a repeated factor). Differences were considered significant at *P*<0.05.

The visible platform and hidden platform versions of the Morris water maze test were conducted to assess spatial learning ability. The apparatus (WM-3002; O'Hara & Co., Ltd, Tokyo, Japan) consisted of a circular tank (30 cm height × 100 cm in diameter) filled with water (22-cm deep) maintained at room temperature (22–24 °C) and made opaque with nontoxic white paint. The surface of the platform (10 cm in diameter) was 1 cm below the water surface. In the visible platform test, six trials per day were conducted for two successive days. There were four possible locations for the platform. The location of the visible platform was changed for every trial. The latency to reach the platform was recorded. In the hidden platform, four trials per day were conducted for six successive days. One of these platform positions was assigned to each mouse as the correct location during the training. The latency to reach the platform, the distance travelled to reach the platform, and average swim speed were recorded. On the sixth day of the training, the platform was removed, and a probe trial was conducted for 60 s. During each probe trial, the time spent in each quadrant, the number of crossings above the former target site, and the average swim speed were recorded. Data acquisition and analysis were performed using Image WM software (O'Hara & Co., Ltd.). The data were statistically analysed using one-way ANOVA or Mann–Whitney *U*-test. Differences were considered significant at *P*<0.05.

The elevated plus-maze (EP-3002; O'Hara & Co., Ltd.) consisted of two open arms (25 × 5 cm) and two enclosed arms of equal size extending from a central area (5 × 5 cm) and elevated 50 cm from the ground. The mice were placed in the central square of the maze facing one of the open arms. Mouse behaviour was recorded during a 10-min test period using a Macintosh computer running Image OFCR × 1.00 and Image OF circle × 1.01 (O'Hara & Co., Ltd.), which is modified from the public-domain code for NIH Image. The following conventional parameters were recorded: the number of entries into open or closed arms and the time spent in open or closed arms. The data were statistically analysed using one-way ANOVA. Differences were considered significant at *P*<0.05.

### Preparation of the Golgi membrane fraction

The preparation of the Golgi membrane fraction was performed using a discontinuous sucrose density gradient, as described previously^48^. Briefly, the total microsomal membranes from the mouse brains were homogenized, adjusted to 1.25 M sucrose, and then layered onto a 1.84 M sucrose cushion. This was overlaid with 1.1 M and 0.25 M sucrose, and the sample was centrifuged at 120,000*g* for 3 h. The fractions were recovered from the 0.25 M/1.1 M interface, the 1.1 M region and the 1.25 M region.

### Cell culture and DNA transfection

SORT1 and TrkB cDNAs, purchased from Origene (MD, USA), were amplified via PCR and subcloned into pME18S expression vectors^7^. The expression plasmid pME-ARHGAP33 was described previously^7^. HEK293T cells were cultured in DMEM+10% FBS and were transfected using TransIT Transfection Reagent (Takara, Ohtsu, Japan) as described previously^12^. Two days later, the cells were collected for protein precipitation. The cells were tested for mycoplasma contamination using PCR.

### Recombinant lentivirus

Recombinant lentivirus was prepared essentially as described previously^11^ with some modifications. Briefly, 5.2 μg of pNHP, 2.1 μg of pHEF-VSVG, 0.43 μg of pCEP4-tat and 2.6 μg of MISSION shRNA construct (TRCN0000034496 and TRCN0000034494, Sigma-Aldrich, MO, USA) were transfected into Lenti-X 293T cells (Clontech, CA, USA) in one 10-cm dish using LF2000 (Invitrogen). After 12 h of incubation, the culture supernatant was replaced with fresh media. After 48 h of incubation, the culture supernatant was collected and stored in aliquots at −80 °C. An shRNA sequence that does not target any mouse genes (SHC002, Sigma-Aldrich) was used as a control. For biochemical analysis, neurons were infected with recombinant lentiviruses at 7 days *in vitro* and maintained for an additional 7 days.

### BDNF immunoassay

The concentration of BDNF secreted in the neuronal culture medium was determined using the Emax Immunoassay system (Promega, WI, USA) according to the manufacturer's protocol. The absorbance was measured using a Bio-Rad iMark microplate reader (Bio-Rad, CA, USA).

### Expression analysis with immortalized lymphoblast

The mRNA expression analysis included 45 Japanese patients with schizophrenia and 45 age- and sex-matched healthy Japanese controls (Supplementary Table 1). Total RNA was extracted from immortalized lymphocytes from the schizophrenia patients and the healthy controls using an RNeasy Mini kit (QIAGEN K.K., Tokyo, Japan). *ARHGAP33* and *SORT1* mRNA expression levels were measured via real-time quantitative RT–PCR using an ABI Prism 7900 sequence detection system (Applied Biosystems), as described previously^54^. The TaqMan Pre-Developed Assay Reagent kit (Applied Biosystems) was used for the mRNA expression analysis of *GAPDH* (4326317E), *ARHGAP33* (Hs00364775_m1) and *SORT1* (Hs00361747_m1). PCR data were obtained using Sequence Detector Software (SDS version 2.1, Applied Biosystems) and were quantified according to a standard curve method. The expression levels of *ARHGAP33* and *SORT1* were normalized to *GAPDH* mRNA.

### Statistical tests

We verified the equality of variance assumption by using the F-test or Levene test. As a pre-test of normality, we used the Kolmogorov–Smirnov test. We applied parametric tests under the assumptions of normality and the equality of variance. Otherwise we used non-parametric tests. The quantified data of the western blots and electrophysiological measurements were statistically analysed using a non-parametric Mann–Whitney *U*-test with the Ryan's correction as necessary for the individual experiments, a non-parametric Kruskal–Wallis test followed by *post hoc* Steel–Dwass tests, or a non-parametric Kolmogorov–Smirnov test. The quantified data of the spine morphology were statistically analysed using a one-way ANOVA or two-way ANOVA, followed by Tukey–Kramer *post hoc* tests. The behavioural data were statistically analysed using parametric tests, including a one-way ANOVA, two-way ANOVA and two-way ANOVA with repeated measures, followed by Tukey–Kramer *post hoc* tests as necessary for the individual experiments, or a non-parametric tests including Mann–Whitney *U*-test with the Ryan's correction and Friedman test followed by Scheffe tests. For details, see the description for each method and figure legend. The differences in the mRNA levels of *ARHGAP33* and *SORT1* between patients and controls were analysed using the Mann–Whitney *U*-test and analysis of covariance, with diagnosis as the independent factor and sex and age as covariates. Spearman's rank order correlation test was performed to assess the possible correlation between *ARHGAP33* and *SORT1* expressions. Two-tailed tests were used, and the significance level was set at *P*<0.05. Statistical analyses were conducted using Stat View software (SAS Institute, NC, USA) or Microsoft Excel with a statistical add-in software (Excel Statistics, SSRI Co., Ltd., Tokyo, Japan).

### Subjects for the genetic association analysis

Subjects for the genetic association analysis included 2,005 unrelated patients with schizophrenia (54.6% males (1,095/910), with a mean age±s.d. of 44.8±15.1 years) and 2,540 unrelated healthy controls (50.2% males (1,274/1,266), 45.4±19.5 years). Our study size of 2,005 patients and 2,540 controls had sufficient power (>80%) to detect a genetic effect at odds ratios (ORs) of 1.146 or greater for rs231228. The mean age did not differ significantly between patients and controls (*P*=0.20); however, the male to female ratio of the patients was significantly higher than in the controls (*P*<0.05). All the subjects used in both analyses were biologically unrelated Japanese and were recruited from four geographical regions in Japan: Osaka, Aichi, Tokushima and Tokyo, which have been reported as regions without population stratification^55^,^56^. The subjects were assessed and diagnosed as described previously^55^,^57^. Briefly, each patient with schizophrenia had been diagnosed by at least two trained psychiatrists by unstructured clinical interviews, according to the criteria of the DSM-IV. Subjects were excluded from this study if they had neurological or medical conditions that could have potentially affected their central nervous system^57^. Psychiatrically healthy controls were evaluated using unstructured interviews to exclude individuals who had current or past contact with psychiatric services. Subjects for brain structure analysis consisted of 124 patients with schizophrenia (56.5% males (70/54), 37.5±12.3 years) and 407 healthy subjects (46.9% males (191/216), 35.4±12.5 years). The details and inclusion criteria of subjects for brain structure analysis have been reported elsewhere^58^. Demographic information for patients with schizophrenia and healthy controls included in the brain structure analyses was shown in Supplementary Table 2. Written informed consent was obtained for all the subjects after the procedures had been fully explained. Confidentiality of all patient information was strictly maintained. All the experiments were blindly performed.

### SNP selection and genotyping

This study was designed to examine the association between the *ARHGAP33* and schizophrenia by selectively tagging SNPs in the *ARHGAP33* (13.3 kb) and flanking regions (±3 kb) located on chromosome 19q13.12. We selected six tagging SNPs using the TAGGER algorithm (Paul de Bakker, http://www.broad.mit.edu/mpg/tagger) with the criteria of *r*^2^ greater than 0.80 in ‘pair-wise tagging only' mode and an MAF greater than 5%, which was implemented in Haploview 4.2 using HapMap data release 24/phaseII Nov 2008, on NCBI B36 assembly, dbSNP b126 [Han Chinese in Beijing, China (CHB)+Japanese in Tokyo, Japan (JPT), Chr 19: 40,955,316–40,974,564]. The six tagging SNPs were rs12982672, rs231228, rs807491, rs231231, rs2291067 and rs120960. Markers are shown in Fig. 8c,d. Venous blood was collected from the subjects and genomic DNA was extracted from whole blood according to standard procedures. The SNPs were genotyped using the TaqMan 5'-exonuclease allelic discrimination assay (Applied Biosystems; Foster City, CA, USA) as previously described^59^,^60^. Genotyping call rates were 98.6% (rs12982672), 99.3% (rs231228), 99.1% (rs807491), 99.2% (rs231231), 98.8% (rs2291067) and 99.0% (rs120960). No deviation from Hardy–Weinberg equilibrium (HWE) in the examined SNPs was detected in the patients with schizophrenia or healthy controls (*P*>0.01).

### Magnetic resonance imaging procedure

The structural images were acquired using a 1.5T GE MRI scanner, and the MRI images were processed using optimized voxel-based morphometry in Statistical Parametric Mapping 5 (SPM5) running on MATLAB R2010b, as previously described^57^. Detailed information regarding the methods is described elsewhere^57^. Statistical analyses were performed with SPM8 software (http://www.fil.ion.ucl.ac.uk/spm/software/spm8/). Whole brain searches to explore the effects of the *ARHGAP33* genotype of risk-rs231228 (individuals with risk T/T genotype versus C carriers) and the genotype–diagnosis interaction on grey matter volume in patients with schizophrenia and healthy subjects were performed. The genotype effect was assessed statistically using a two-sample *t*-test model. The genotype–diagnosis interaction on the grey matter volume was assessed full factorial model with diagnosis as a factor and genotype status as a covariate interacted with the diagnosis. Age, sex and years of education were included as covariates of no interest into the analyses to control for confounding variables. A non-sphericity correction was used for all analyses. These analyses yielded statistical parametric maps (SPM) based on a voxel-level height threshold of *P*<0.001 (uncorrected for multiple comparisons). Clusters of more than 100 contiguous voxels were considered in the analyses. Small volume correction (SVC) was applied to protect against type I error using family-wise error. The significance level was set as *P*<0.05 (family-wise error corrected) after SVC for spheres with a radius of 10 mm around the peak. Volume-of-interest (VOI) approach was performed to further compare significant regions of diagnosis–genotype interaction in the whole-brain analyses. We extracted a sphere of 10 mm VOI radius from regions of interest because SVC applied for spheres with a radius of 10 mm around the peak. Anatomic localization was presented as Talairach coordinates.

### Statistical tests for the genetic association analysis

Statistical analyses of demographic variables were performed using PASW Statistics 18.0 software (SPSS Japan Inc., Tokyo, Japan). Differences in clinical characteristics between patients and controls or between genotypes were analysed using *χ*^2^ tests for categorical variables and a Mann–Whitney *U*-test for continuous variables. The presence of HWE and the allelic distributions of *ARHGAP33* polymorphisms between patients and controls were analysed by the *χ*^2^ test using SNPAlyze V5.1.1 Pro software (DYNACOM, Yokohama, Japan), and the significance level for HWE was set at *P*<0.01. The Bonferroni correction for multiple testing was applied to protect against type I error. Our tested SNPs were six and corrected *P* value for allelic associations was set at *P*<0.0083. Relative grey matter volumes as the ‘y' values from maxima voxel in the region of interest were extracted and used in the VOI analysis using PASW. The effects of the *ARHGAP33* genotype on the extracted VOI were tested using ANOVAs without covariates, because the extraction of VOI was performed after confounding factors including age, sex and education years were included in the whole-brain analysis. The two-tailed tests were used, and the significance level was set at *P*<0.05.

## Additional information

**How to cite this article:** Nakazawa, T. *et al*. Emerging roles of ARHGAP33 in intracellular trafficking of TrkB and pathophysiology of neuropsychiatric disorders. *Nat. Commun.* 7:10594 doi: 10.1038/ncomms10594 (2016).

## Supplementary Material

Supplementary Information Supplementary Figures 1-5, Supplementary Tables 1-3 and Supplementary Methods

## Figures and Tables

**Table 1:** Allelic frequencies of SNPs in the *ARHGAP33* among schizophrenia patients and controls.

**Marker**		**M/m**	**Gene**	**MAF**	**Allelic**	***P*** (***χ***^**2**^)	**OR (95% CI)**
**SNP IDs**	Position*			**SCZ (*****n*****=2,005)**	**CON (*****n*****=2,540)**		
rs12982672	40960311	G/A	Intron1	0.11	0.10	**0.021 (5.3)**	1.17 (1.02–1.34)
rs231228	40960611	C/T	Exon3	0.26	0.23	**0.0064 (7.4)**	1.14 (1.04–1.26)
rs807491	40960763	T/C	Intron3	0.43	0.44	0.28 (1.2)	0.96 (0.88–1.04)
rs231231	40961892	G/A	Intron6	0.30	0.30	0.85 (<0.1)	0.99 (0.91–1.09)
rs2291067	40965374	G/A	Exon14	0.16	0.16	0.59 (0.3)	1.03 (0.92–1.15)
rs120960	40968607	G/A	Intron19	0.13	0.13	0.73 (0.1)	1.02 (0.90–1.16)

CI, confidence interval; CON, controls; m, minor allele; M, major allele; MAF, minor allele frequency; OR, odds ratio; SCZ, schizophrenia patient.

^*^db SNP build 129. All the alleles are represented according to the minus strand DNA sequence. *P* values <0.05 are in bold and underlined.
