# Corrigendum: Emerging roles of ARHGAP33 in intracellular trafficking of TrkB and pathophysiology of neuropsychiatric disorders

**DOI:** 10.1038/ncomms11466

**Published:** 2016-04-20

**Authors:** Takanobu Nakazawa, Ryota Hashimoto, Kazuto Sakoori, Yuki Sugaya, Asami Tanimura, Yuki Hashimotodani, Kazutaka Ohi, Hidenaga Yamamori, Yuka Yasuda, Satomi Umeda-Yano, Yuji Kiyama, Kohtarou Konno, Takeshi Inoue, Kazumasa Yokoyama, Takafumi Inoue, Shusuke Numata, Tohru Ohnuma, Nakao Iwata, Norio Ozaki, Hitoshi Hashimoto, Masahiko Watanabe, Toshiya Manabe, Tadashi Yamamoto, Masatoshi Takeda, Masanobu Kano

Nature Communications 7: Article number:1059410.1038/ncomms10594 (2016); Published: 02032016; Updated: 04202016

Previous work by Rosário *et al*. 2007 and Schuster *et al*. 2015 describing the role of ARHGAP33 in regulating dendrite and dendritic spine development, as well as neuropsychiatric disorder-related behaviours in developing and adult mice, was inadvertently omitted from the reference list of this Article and should have been cited as follows. The statement in the Results section ‘The roles of ARHGAP33 in the adult brain have not been investigated' should have read ‘Previous work has investigated the role of ARHGAP33 in dendrite and dendritic spine development, as well as in autism-like behaviours (Rosário *et al*. 2007, Schuster *et al*. 2015, refs 6–9)'.

Rosário, M., Franke, R., Bednarski, C., & Birchmeier, W. The neurite outgrowth multiadaptor RhoGAP, NOMA-GAP, regulates neurite extension through SHP2 and Cdc42. *J. Cell. Biol*. **178**, 503–516 (2007).

Schuster, S., *et al*. NOMA-GAP/ARHGAP33 regulates synapse development and autistic-like behavior in the mouse. *Mol. Psychiatry* **20**, 1120–1131 (2015).

