# Supplementary material for: Emerging roles of ARHGAP33 in intracellular trafficking of TrkB and pathophysiology of neuropsychiatric disorders
Source: Nat Commun. 2016 Feb 3;7:10594. doi: 10.1038/ncomms10594 (PMC4742909; doi:10.1038/ncomms10594)
Supplement: Supplementary Information — Supplementary Figures 1-5, Supplementary Tables 1-3 and Supplementary Methods [file ncomms10594-s1.pdf]

Supplementary Figures

Supplementary Figure 1

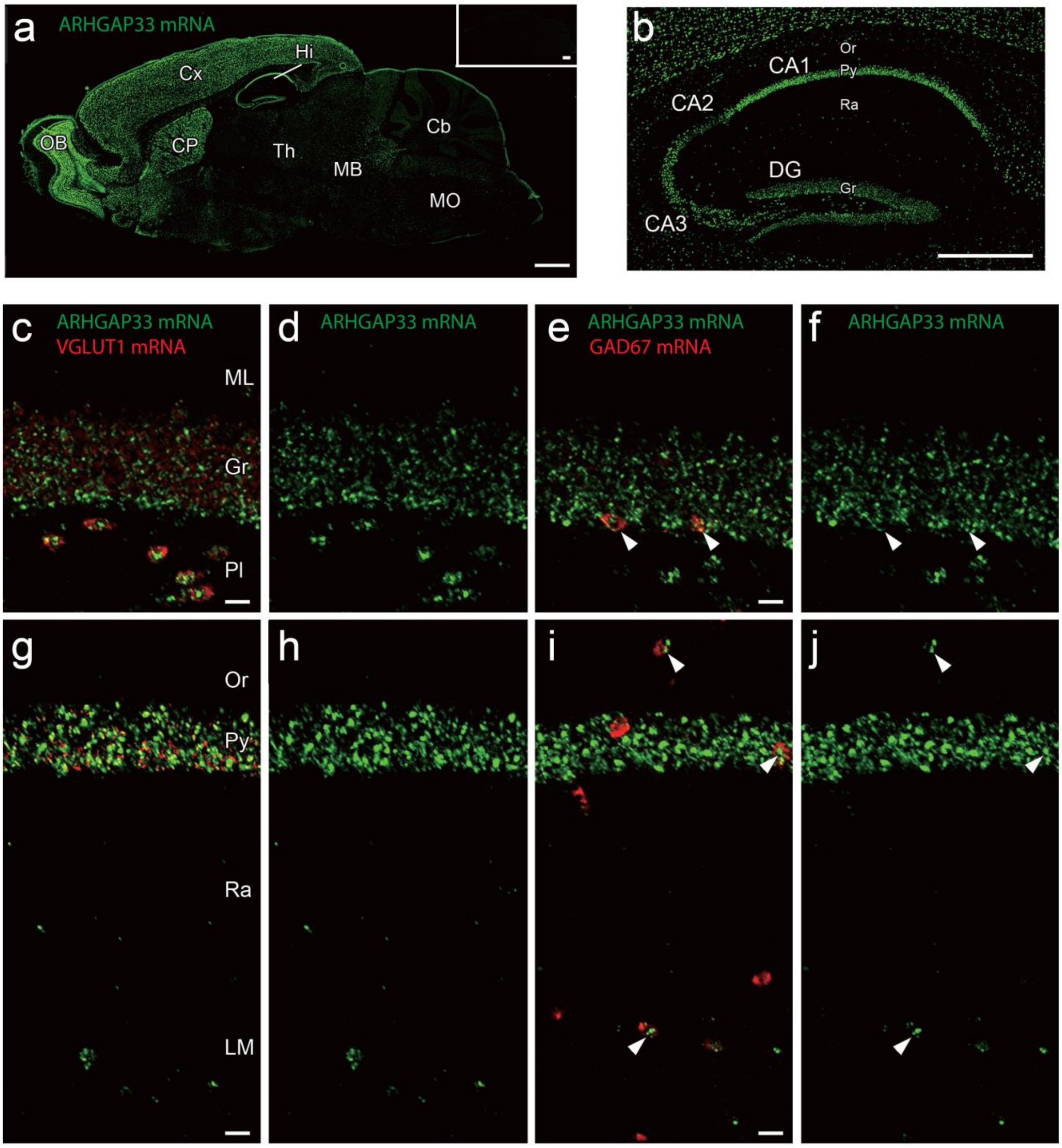

Supplementary Figure 1 *ARHGAP33* mRNA localization.

(**a**, **b**) Fluorescent *in situ* hybridization for *ARHGAP33* mRNA in a sagittal section of adult WT mice (**a**) and *ARHGAP33* KO mice (**a**, inset). Higher magnification of the hippocampus is shown in **b**. Cb, cerebellum; CP, caudate-putamen; CX, cerebral cortex; Hi, hippocampus; MB, midbrain; MO, medulla oblongata; OB, olfactory bulb; Th, thalamus. CA, cornu ammonis; DG, dentate gyrus; Gr, granule cell layer; Or, oriens layer; Py, pyramidal cell layer; Ra, radiatum layer. Scale bars, 1 mm (**a**), 500 µm (**b**). (**c-f**) Fluorescent *in situ* hybridization for *ARHGAP33* mRNA together with *VGLUT1* mRNA (**c**) and *GAD67* mRNA (**e**) in the dentate gyrus of the hippocampus. Gr, granule cell layer; ML, molecular layer; Pl, polymorphic layer. Scale bars, 20 µm. (**g-j**) Fluorescent *in situ* hybridization for *ARHGAP33* mRNA together with *VGLUT1* mRNA (**g**) and *GAD67* mRNA (**i**) in the CA1 region of the hippocampus. LM, stratum lacunosum-moleculare; Or, Stratum oriens; Py, pyramidal cell layer; Ra, stratum radiatum. Scale bars, 20 µm. Note that *ARHGAP33* mRNA was colocalized both with *VGLUT1* mRNA and *GAD67* mRNA (arrow head) in the dentate gyrus and the CA1 region of the hippcampus (**c**, **e**, **g**, **i**).

Supplementary Figure 2

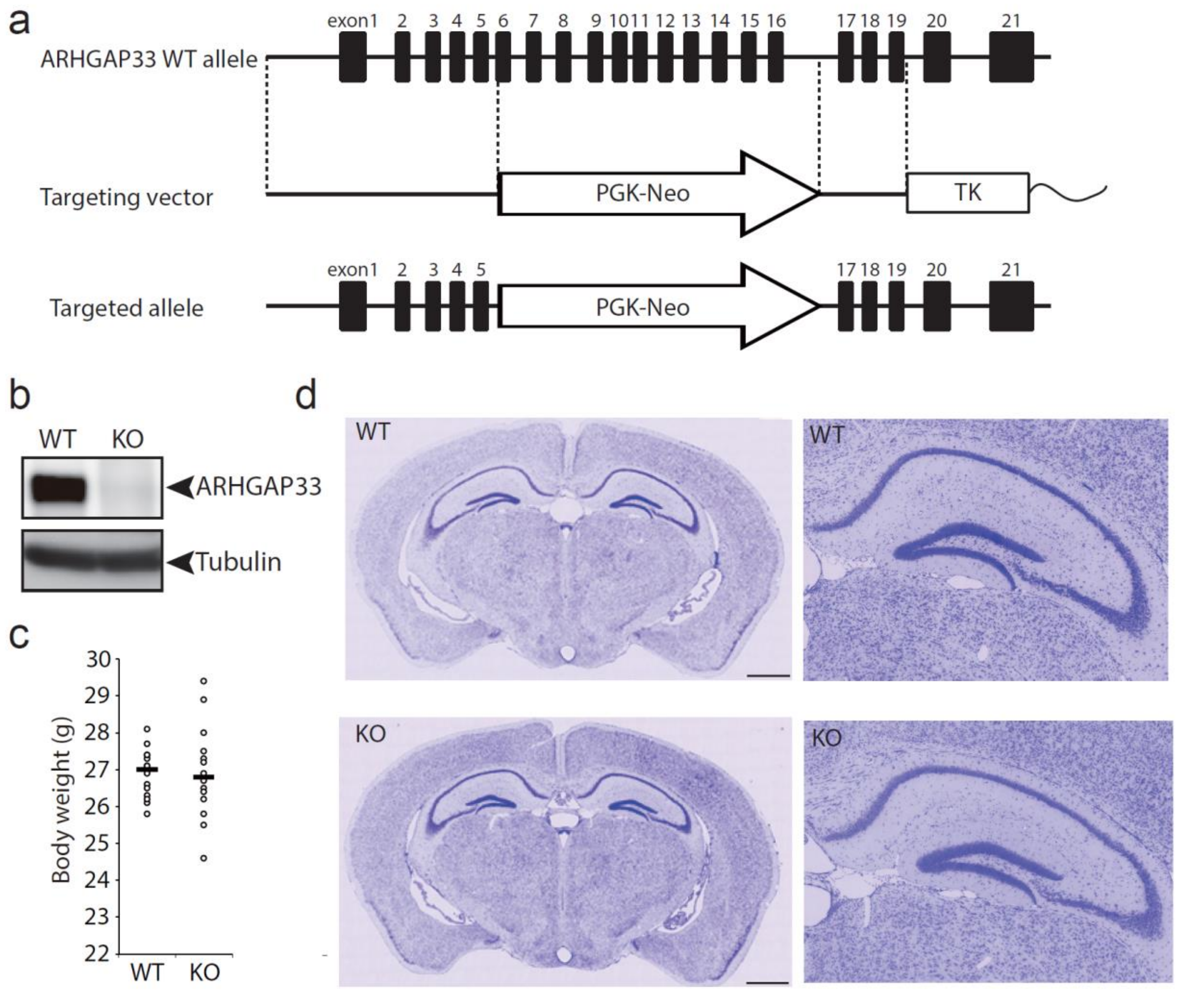

Supplementary Figure 2 Generation of *ARHGAP33* KO mice.

(**a**) Schematic representation of the wild-type allele, targeting vector, and the targeted *ARHGAP33* allele. Neo, neomycin-resistant gene; PGK, phoshoglycerate kinase promoter; TK, thymidine kinase gene. (**b**) Immunoblotting for ARHGAP33 in adult wild-type and *ARHGAP33* KO mouse brain lysates. Tubulin was used as a loading control. (**c**) Normal body size of *ARHGAP33* KO mice compared to WT mice (WT, $N = 14$; *ARHGAP33* KO, $N = 14$; $P > 0.05$, Mann-Whitney U-test). Bars show median values. (**d**) Nissl staining in coronal brain sections including hippocampus of adult WT and *ARHGAP33* KO mice. Higher magnifications of the hippocampus are shown in the right panels. Scale bars, 1 mm.

Supplementary Figure 3

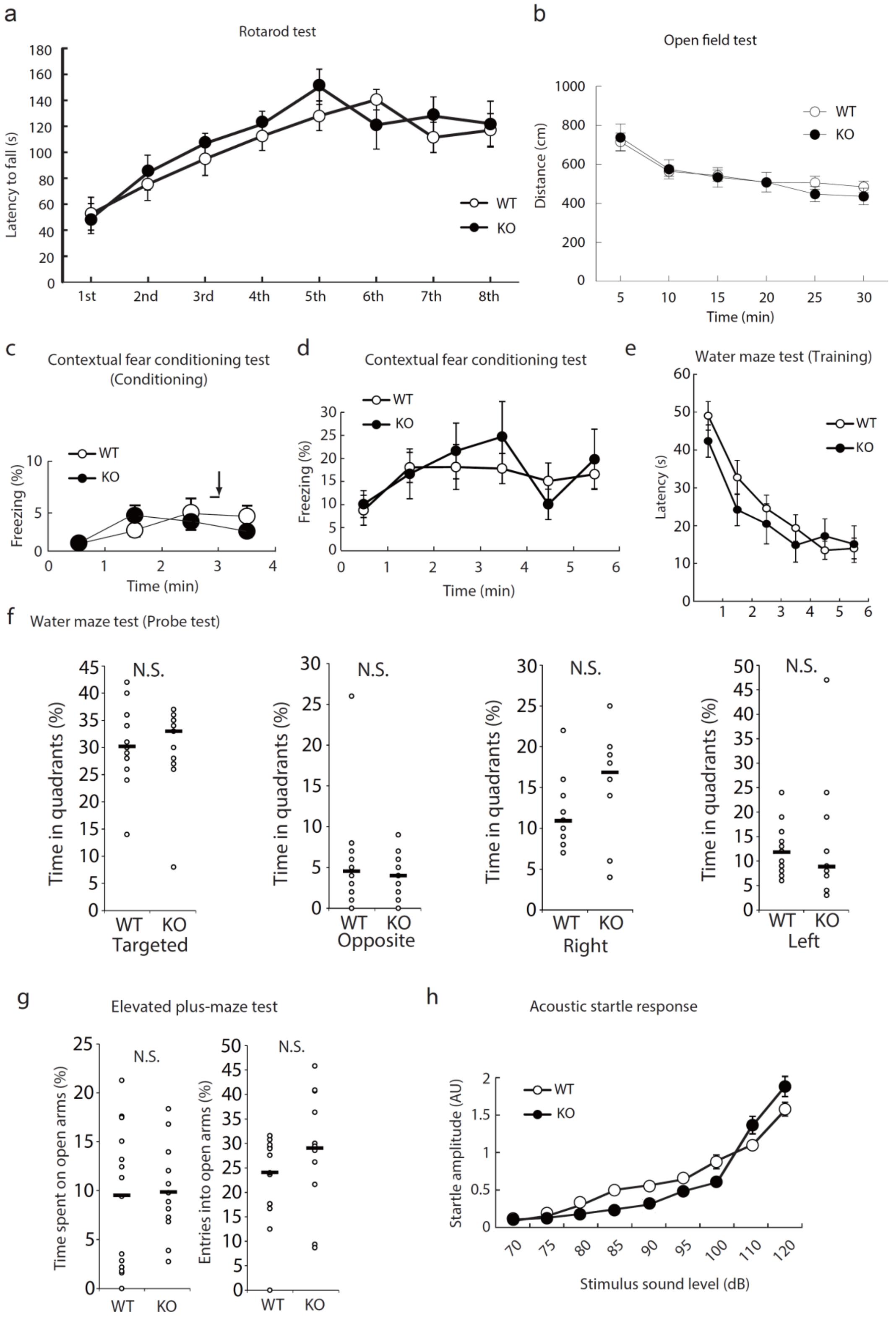

Supplementary Figure 3 Normal behavior of *ARHGAP33* KO mice.

(**a**) The rotarod test. The average of time spent on the rotarod across 8 test trials for WT (open circles) and *ARHGAP33* KO (closed circles) mice (WT, N = 12, *ARHGAP33* KO, N = 11). There were no significant differences between the two genotypes ($P > 0.05$, repeated-measures ANOVA). The data are expressed as mean ± s.e.m. (**b**) The open field test. Normal locomotor activity in *ARHGAP33* KO mice (closed circles) in the open field test compared to WT mice (open circles) (WT, N = 14, *ARHGAP33* KO, N = 14). There were no significant differences between the two genotypes ($P > 0.05$, Two-way ANOVA with repeated measures). The data are expressed as mean ± s.e.m. (**c**, **d**) The contextual fear conditioning test. Freezing responses on the conditioning day (**c**). 170 s after the placement of mice in the conditioning chamber, a tone was presented for 10 s (solid line); at the end of the tone, mice were given a footshock for 2 s (arrow). Freezing responses in the contextual fear conditioning test (WT, N = 14, *ARHGAP33* KO, N = 12, $P > 0.05$, Two-way ANOVA with repeated measures) (**d**). There were no significant differences between the two genotypes ($P > 0.05$, Two-way ANOVA with repeated measures). The data are expressed as mean ± s.e.m. (**e**, **f**) The Morris water maze test. The escape latency in the training session (WT, N = 14, *ARHGAP33* KO, N = 12, $P > 0.05$, Two-way ANOVA with repeated measures) (**e**). The time spent in each quadrant during the probe trials (WT, N = 14, *ARHGAP33* KO, N = 12, $P > 0.05$, Mann-Whitney U-test) (**f**). Bars show median values. (**g**) The elevated plus-maze test. The time spent on open arms (WT, N = 14, *ARHGAP33* KO, N = 12, $P > 0.05$, one-way ANOVA) (left). Entries into open arms (WT, N = 14, *ARHGAP33* KO, N = 12, $P > 0.05$, one-way ANOVA) (right). Bars show mean values. (**h**) Responses to acoustic stimuli. Mean amplitudes of startle responses in WT (open circles) and *ARHGAP33* KO (closed circles) mice (WT, N = 12, *ARHGAP33* KO, N = 11) are shown. There were no significant differences between the two genotypes ($P > 0.05$, Two-way ANOVA with repeated measures). The data are expressed as mean ± s.e.m. N.S., not significant.

Supplementary Figure 4

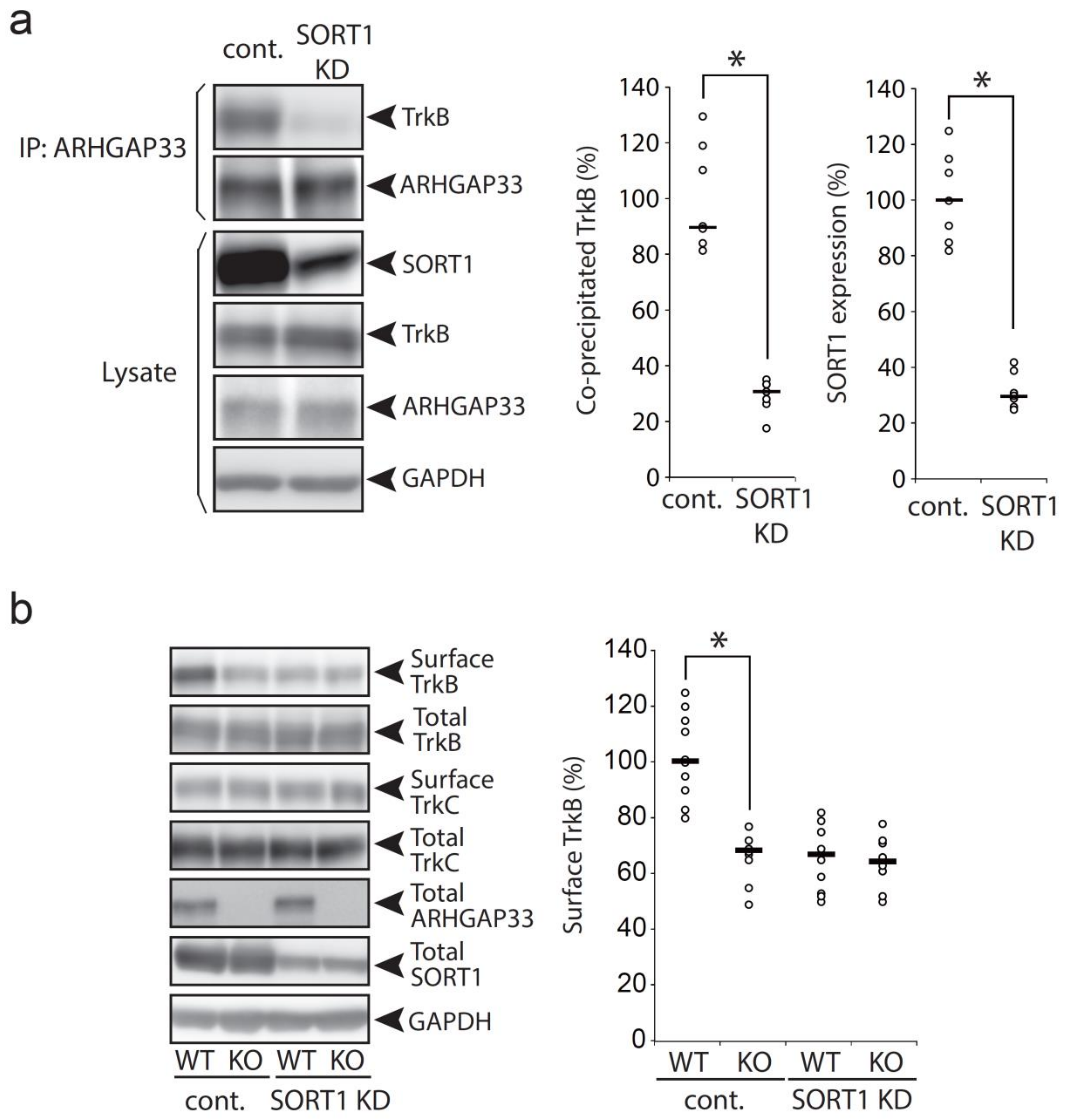

Supplementary Figure 4 Effects of SORT1 knockdown with shRNA construct (TRCN0000034494).

(**a**) Weakened interaction between ARHGAP33 and TrkB in the SORT1 knockdown neuron. ARHGAP33 immunoprecipitates and total lysates were immunoblotted with the indicated antibodies. Representative blots (left), quantification of co-immunoprecipitated TrkB (center), and quantification of SORT1 expression (right)

(each N = 7; TrkB, P = 0.0017, SORT1, P = 0.0017, Mann-Whitney U-test). The averaged values of the control neurons were set to 100%. Bars show median values. Western blots show representative results from 7 independent experiments performed using neurons from different mice. cont., control. KD, knockdown. Note that another shRNA construct (TRCN0000034494) targeted to region of SORT1 that does not overlap with that targeted by the shRNA used in Fig. 7c was used. (**b**) Requirement of SORT1 in ARHGAP33-mediated TrkB trafficking. Biotinylated cell surface proteins and total lysates were immunoblotted with the indicated antibodies. Representative blots (left) and quantification of surface TrkB expression (right) (each N = 10; WT vs *ARHGAP33* KO in the control neurons, corrected $P = 6.2\times10^{-4}$; WT vs *ARHGAP33* KO in the SORT1 knockdown neurons, corrected $P > 0.05$, Mann-Whitney U-test with the Ryan's correction). The averaged values of the control neurons from WT mice were set to 100%. Western blots show representative results from 10 independent experiments performed using neurons from different mice. cont., control. KD, knockdown. Bars show median values. Note that another shRNA construct (TRCN0000034494) targeted to region of SORT1 that does not overlap with that targeted by the shRNA used in Fig. 7c was used.

Supplementary Figure 5

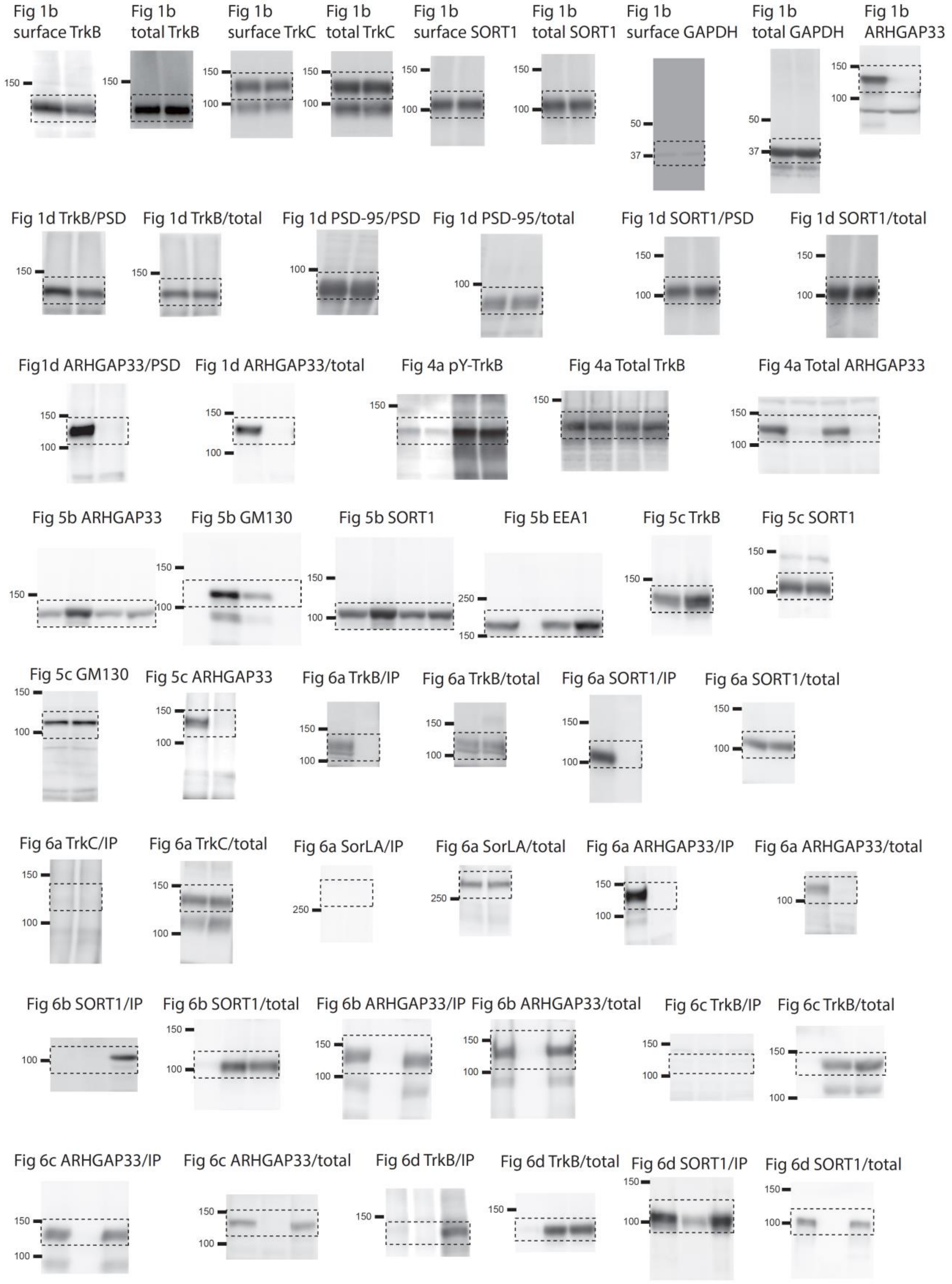

Supplementary Figure 5 Original uncropped images of western blots.

Supplementary Figure 5 (continued)

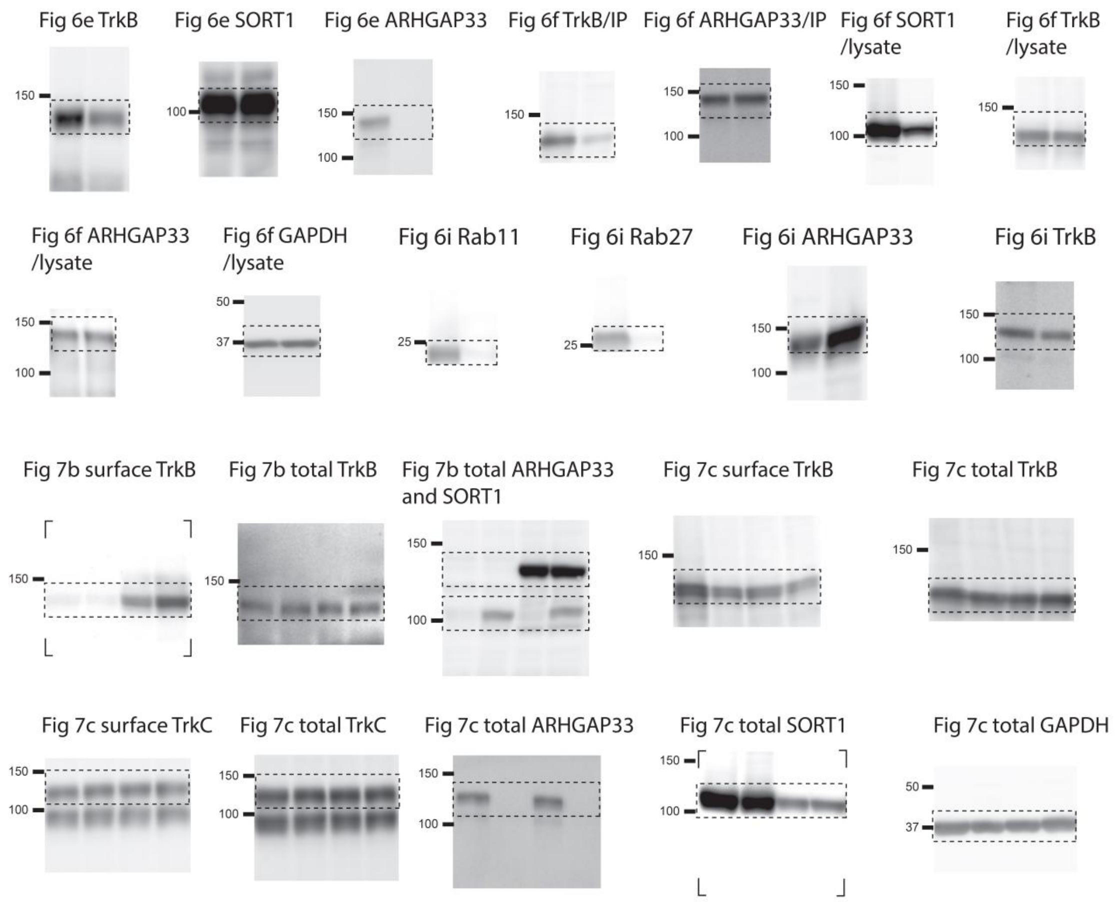

Supplementary Figure 5 (continued) Original uncropped images of western blots.

Supplementary tables

Supplementary Table 1. Demographic information for patients with schizophrenia and healthy controls included in expression analysis

Variables	Schizophrenia (*N* = 45)	Control (*N* = 45)
Age (years)	32.3 ± 10.7	32.3 ± 12.7
Sex (male/female)	29/16	29/16
CPZ-eq (mg/day)	737.7 ± 587.1	-
Age at onset (years)	22.2 ± 8.5	-
Duration of illness (years)	9.9 ± 7.8	-
PANSS Positive Symptoms	24.0 ± 7.3	-
PANSS Negative Symptoms	25.8 ± 8.6	-
PANSS General Psychopathology	54.3 ± 14.1	-

PANSS, Positive and Negative Syndrome Scale;

CPZ-eq, chlorpromazine equivalent of total antipsychotics. Mean ± s.d.

Supplementary Table 2. Demographic information for patients with schizophrenia and healthy controls included in brain structure analysis

Variables	Schizophrenia (*N* = 124)	Control (*N* = 407)	*P* values (*z*)
Age (years)	37.5 ± 12.3	35.4 ± 12.5	0.055 (1.9)
Sex (male/female)	70/54	191/216	0.063 (3.4)[a]
Education (years)	14.1 ± 2.3	15.0 ± 2.2	**<u><0.001 (-3.2)</u>**
Estimated premorbid IQ	101.9 ± 10.1	107.6 ± 8.1	**<u><0.001 (-5.6)</u>**
Handedness (Rt./Lt./Bil.)	120/4/0	383/23/1	0.48 (1.5)[a]
CPZ-eq (mg/day)	572.8 ± 536.4	-	-
Age at onset (years)	24.9 ± 10.2	-	-
Duration of illness (years)	12.5 ± 9.8	-	-
PANSS Positive Symptoms	18.5 ± 5.7	-	-
PANSS Negative Symptoms	19.3 ± 6.2	-	-
PANSS General Psychopathology	40.6 ± 11.1	-	-

PANSS, Positive and Negative Syndrome Scale; CPZ-eq, chlorpromazine equivalent of total antipsychotics. Mean ± s.d. and *p* values are shown. Significant *P* values are shown as bold face and underlined. [a]χ^2 test. Complete demographic information was not obtained for all subjects (estimated premorbid IQ and PANSS in patients: *N* = 115 and *N* = 122; estimated premorbid IQ in controls: *N* = 406).

Supplementary Table 3. Effects of *ARHGAP33* genotype-diagnosis interaction on brain structure in total subjects

Brain regions	R/L	BA	CS	*T*	corrected *p* value	Talairach coordinates		
						x	*y*	*z*
***ARHGAP33* genotype-diagnosis interaction**								
Middle Temporal Gyrus	L	21	286	4.39	7.2×10^{-4}	-49	6	-35
Medial Frontal Gyrus	R	25	555	4.26	1.1×10^{-3}	5	16	-13
Inferior Temporal Gyrus	R	20	451	3.69	8.5×10^{-3}	57	-12	-20

R, right; L, left; BA, Brodmann area; CS,Cluster size; T, Peak-voxel T.

Supplementary Methods

Nucleotide sequence of the cRNA probe of *ARHGAP33* for *in situ hybridization*

5′-
ATGCTCCAGA CACAGAGAGA GTCAGATCCC ATCCTGCCTT GGGGAGCTTC ATGGGCTGGC AGGGGACAGA CCCTGAGGGC CCGAAGCACT
GACAGCCTGG ATGGCCCAGG GGAGGGCTCA GTGCAGCCTG TTCCCTCTAC TGGAGGGCCC AGTGTGAAGG GGAAGCCTGG GAAGAGGCTC
TCAGCTCCTC GAGGTCCCTT TCCTCGGCTG GCCGACTGTG CCCATTTCCA CTATGAGAAT GTTGACTTTG GCCATATTCA GCTCCTACTG
TCTCCAGAGC GTGAAGGCCC CAGCCTCTCT GGAGAGAATG AGCTGGTATT TGGGGTGCAG GTAACCTGTC AGGGCCGTTC TTGGCCAGTT
CTTCGTAGTT ACGATGACTT CCGTTCCCTG GATGCCCACC TGCACCGATG CATATTTGAC CGGAGGTTTT CCTGCCTTCC AGAGCTCCCT
CCACCCCCAG AGGGTGCTAG GGCTGCCCAG ATGCTGGTAC CTCTGCTGCT GCAGTATCTG GAGACCCTGT CTGGACTGGT GGACAGTAAC
CTCAACTGTG GGCCTGTGCT CACTTGGATG GAGCTGGACA ATCATGGCCG ACGATTGCTC CTCAGTGAGG AGGCCTCCCT CAATATCCCT
GCAGTGGCTG CCGCTCATGT GGTCAAACGG TACACAGCTC AGGCACCAGA TGAGCTCTCC TTTGAGGTGG GAGACATCGT CTCAGTGATC
GACATGCCAC CCACAGAGGA TCGGAGCTGG TGGCGGGGCA AGCGGGGCTT CCAGGTTGGT TTCTTCCCCA GCGAGTGTGT AGAACTCTTC
ACAGAGAGGC CAGGTCCTGG CTTAAAGGCA GATGCTGATG GTTCCCTGTG TGGCATCCCA GCTCCCCAGG GTAACTCTTC TCTCACCTCA
GCTGTGCCCC GGCCACGTGG GAAGCTGGCT GGCCTCCTCC GAACCTTCAT GCGCTCCCGC CCTTCTCGGC AGCGGTTGCG GCAGCGGGGA
ATTCTGCGGC AGAGAGTATT TGGCTGTGAC CTTGGAGAGC ATCTCAGCAA CTCAGGCCAG GATGTGCCCC AAGTGCTGCG CTGCTGCTCT
GAGTTTATTG AGGCCCATGG GGTGGTGGAT GGAATCTACC GGCTCTCAGG CGTGTCCTCC AACATCCAGA GGCTACGGCA TGAATTTGAC
AGTGAGAGGA TTCCTGAACT ATCTGGCCCT GCCTTCCTGC AGGACATCCA CAGTGTATCC TCCCTTTGCA AACTCTACTT CCGAGAGCTA
CCAAACCCCC TACTCACCTA CCAGCTCTAT GGGAAATTCA GTGAGGCCAT GTCAGTCCTA GGGGAGGAAG AACGCCTGGT GAGAGTCCAT
GATGTCATCC AGCAGTTGCC CCCACCACAC TACAGGACTC TAGAGTACCT GCTGAGGCAC TTGGCCCGCA TGGCAAGACA TAGTGCCAAT
ACCAGCATGC ATGCCCGCAA CTTGGCCATT GTCTGGGCAC CCAACCTGCT CCGGTCCATG GAGCTAGAGT CAGTGGGGCT GGGTGGGGCG
GCAGCCTTCC GGGAGGTTCG TGTGCAGTCA GTGGTGGTGG AATTCCTGCT CACCCATGTG GAGGTCCTGT TTAGTGACAC CTTCACCTCT
GCTGGCCTAG ACCCTGCAGG CCGCTGTCTT CTCCCCAGGC CCAAGTCCCT TGCGGGAAGT GGCCCCTCTA CTCGCCTGCT GACACTGGAA
GAAGCCCAGG CTCGTACCCA GGGCCGGCTG GGAACACCCA CTGAGCCCAC AACTCTCAAG GCTGCAGCCT CACCTGTGGA AAAGAGGAAA
AGGGAGAGAG GGGAAAAACA GCGGAAGCCA GGGGGCAGCA GCTGGAAGAC ATTCTTTGCT CTTGGCCGGG GCCCCAGCAT CCCCCGGAAG
AAGCCTCTAC CCTGGCTGGG GGGCTCTCGT GCACCACCAC AGCCTTCAGG CAACCGACCT GACACAGTCA CACTGAGGTC GGCTAAGAGT
GAAGAGTCTC TATCATCACA GGCCAGTGGG ACTGGCCTCC AGAGGCTACA CAGGCTACGG CGACCCCACT CCAGCAGCGA TGCTTTCCCA
GTGGGCCCAG CACCTGCTGG CTCCTGCGAG ACCCTGTCTT CTTCTTCTTC TTCTTCCTCC TCCTCGTCAG CCTCCTCCTC ATCATCATCA
TCTGAGTCCT CAGCAGCTGG GCTGGGGCCA CTTTCCGGGT CACCCTCACA TCGCACCTCA GCCTGGCTAG ATGATGGTGA TGAACTGGAC
TTTAGCCCAC CCCGCTGCCT GGAGGGACTC CGGGGCCTGG ACTTTGATCC GCTTACCTTT CGCTGCAGCA GCCCCACCCC AGGGGACCCT
GCACCTCCTG CCAGCCCAGC ACCCCCAGCA CCCGCCTCTG CCTGCCCACC CAGGGCAACC CCCAAGGCTC CCTCACCCCG AGGACCCACC
AGCCCAGCTT CGTCCACTGC TCTGGACATC TCAGAGCCCC TGGCTGTGTC TGTACCACCT GCTGTCCTAG AACTGCTGGG GGCAGGAGGA
ACACCTGCCT CGGTCACCCC AACACCAGCT CTCAGCCCTG GCCCAGGCTT GCGCCCCCAT CTCATCCCCT TGCTGCTGCA TGGATCAGAG
GCCCAGCTAA GTGACACCTG CCAACAAGAG ATCAGCAGTA AGCTGGCACT GCCTGGTCCT GGGGGAAGCC AAGGTCCTGG TATAGATTCA
CCATTGCTGC CCCCACCTCT GTCCCTTCTA CGCCCTGGTG GGGCCCCACC CCCACCCCCC AAGAACGCAG CGCGCCTCAT GGCACTGGCC
CTGGCTGAGC GGGCTCAGCA GGTGGCTAAG CAACAGAGCC AGCAGGAGCA TGCAAGCACC TCAACTAGTC CACACTCCCC TTTCCGCCGT
TCACTGTCCC TGGAGGTCGG TGGGGAACCT GTGGTAACTT CAGGGAGTGG ACCACCCCCT CACTCCCTAG CCCACCCTGG TGTCTGGGCC
CCAGGACCCC CACCCTACCT ACCAAGGCAA CAAAGTGATG GGAGCCTGGT ACGGAGCCAG CGGCCTGTGG GGACCTCAAG GAGGGGTCTC
AGAGGCCCTG CTCAGGTTCC TACCCCTAGT GTCTTTTCTC CAGCTCCCCG GGAGTGCCTG CCACCTTTCC TTGGGGTCTC CAAACCAGGC
TTGTACCCTC TCGCCCCCTC CTCCTTTCAA CCCAACTCCT CAGCCCAAGT CTGGAGGAAT TCTCTGGGCC CCCCTGCACC TCTTGACAGG
GGAGAGAACC TGTACTATGA GATTGGGGCT AGTGAGGGTT CCCCCTATTC TGGGCCCACT CGGTCCTGGA GTCCTTTTCG CTCCATGCCC
CCTGATAGGC TCAGTGCCTC ATATGGCATG CTTGGCCAGT CACCACCACT TCACAGGTCA CCTGACTTCC TGCTCAGCTA CCCACCGCCC
CCCTCCTGCT TTCCACCTGA CCACCTTGGC TATTCGGTCC CCCAGCACCC TGCCCGGCAC CCTACCCGGC CAGAGCCCCT CTATGTCAAC
CTAGCCCTAG GGCCCAGGGG TCCTTCACCT GCCTCTTCCT GCTCCTCTTC CCCTCCTGCC CATCCCAGAA GTCGTTCGGA TCCTGGGCCC
CCAGCACCTC GTCTCCCTCA GAAGCAGCGG GCTCCCTGGG GCTCCAATAC CCCTCATAGG GTAACAGGAC CTTGGGGCCC TCCTGAACCT
CTCCTGCTCT ACAGGGGATC CCCACCAGCC TATGGGAGGG GGAGCGAGCA TTACCAAGGG TCTTTGTACA GAAATGGGGG TCAGAGGGGA
GAGGGGGCTG GTCCTCCACC TCCTTACCTC ACTCCCAGTT GGTCCCTCCA CTCTGAGGGC CAGACCCGAA GCTACTGTTG A-3′
